# The Length of a Ubiquitin Chain: A General Factor for Selective Recognition by Ubiquitin‐Binding Proteins

**DOI:** 10.1002/anie.202003058

**Published:** 2020-06-08

**Authors:** Joachim Lutz, Eva Höllmüller, Martin Scheffner, Andreas Marx, Florian Stengel

**Affiliations:** ^1^ Departments of Chemistry and Biology Konstanz Research School Chemical Biology University of Konstanz Universitätsstrasse 10 78457 Konstanz Germany

**Keywords:** chain length, posttranslational modification, Ub-binding proteins, ubiquitin

## Abstract

The attachment of ubiquitin (Ub) chains of various length to proteins is a prevalent posttranslational modification in eukaryotes. The fate of a modified protein is determined by Ub‐binding proteins (UBPs), which interact with Ub chains in a linkage‐selective manner. However, the impact and functional consequences of chain length on the binding selectivity of UBPs remain mostly elusive. We have generated Ub chains of defined length and linkage by using click chemistry and GELFrEE fractionation. These defined polymers were used in affinity‐based enrichment assays to identify length‐ and linkage‐selective interaction partners on a proteome‐wide scale. For the first time, it is revealed that the length of a Ub chain generally has a major impact on its ability to be selectively recognized by UBPs.

## Graphical abstract

**The long and the short of it**: An efficient approach has been established to separate homogeneous, triazole‐linked ubiquitin (Ub) chains. Affinity‐based proteomic profiling allowed the identification of length‐selective interaction partners of K27‐, K29‐, and K33‐linked chains, thereby showing the implications of chain length on Ub chain recognition.

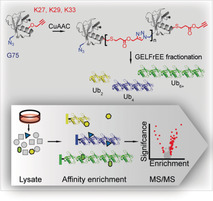

The covalent attachment of ubiquitin (Ub) to proteins (ubiquitylation) is one of the most complex and versatile posttranslational modifications in eukaryotes. It is mediated by the concerted action of three classes of enzymes,^1^, ^2^ which link Ub to substrate proteins through the formation of an isopeptide bond between its C‐terminal carboxy group and the ϵ‐NH_2_ group of a lysine residue in the substrate. In addition to the attachment of single Ub moieties (mono‐ubiquitylation), Ub itself can serve as a substrate, thereby resulting in the formation of Ub chains (poly‐ubiquitylation) that adopt different conformations depending on which of the seven lysine residues (K6, K11, K27, K29, K33, K48, K63) or the N‐terminal methionine residue is used for bond formation. Moreover, Ub chains can be grouped into homotypic, heterotypic, and branched chains.1b Thus, proteins can in theory be modified by an almost infinite number of different Ub chains. K48‐linked Ub chains are the predominant signal for proteasomal degradation, whereas K63‐linked polymers are mainly involved in nondegradative functions affecting processes such as DNA repair and NF‐κB signaling.1b Less functional information is available for the remaining five homotypic K‐linked chains. Of those, K27‐linked chains have been allocated functions in mitochondrial maintenance, mitophagy, protein secretion, and autophagy.^3^, ^4^ K33‐linked chains have been connected to different biological processes, including TCR signaling or, together with K29‐linked chains, the regulation of AMPK‐related protein kinases (AMPK=AMP‐activated protein kinase).3a Furthermore, K29‐linked chains are likely to also serve as a signal for the proteasome.^5^

Similar to the linkage, the length of a Ub chain may represent another, even less understood, determinant of Ub chain recognition. It has been shown that a minimum length of *n*=4 is in many cases required for K48‐linked chains to constitute an effective degradation signal.^6^ However, recent studies indicate that the proteasomal Ub signal is adaptive and that, in certain cases, shorter polymers are also able to promote proteasomal degradation of a substrate.^7^ Another example of the potential significance of the chain length is the deubiquitylating enzyme (DUB) USP5, which harbors an ensemble of four UBDs (UBD=ubiquitin binding domain) and binds to Ub tetramers with much higher affinity than to dimers,^8^ as well as the DUB UCH‐L3, which preferentially cleaves shorter chains over longer polymers.^9^ These examples provide a first hint that the length of a Ub chain adds another important layer of complexity to the Ub code. However, likely because of the lack of proper tools, there has been no study published to date that addresses the general relevance of chain length for Ub chain recognition on a proteome‐wide level. We and others recently generated various methods for studying proteome‐wide interactions with linkage‐defined Ub chains.^10^ In brief, non‐hydrolyzable, triazole‐linked Ub chains were used as bait molecules for affinity purification mass spectrometry (AP‐MS) was used to identify linkage‐specific interaction partners. NMR spectroscopy and modeling studies revealed that the triazole linkage is a reliable surrogate of the natural isopeptide bond.^11^ However, the potential dependency of such interactions on the Ub chain length wasnot addressed in previous studies. This is mainly due to the lack of sufficient amounts of length‐defined Ub chains, despite remarkable progress in the chemical synthesis and semisynthesis of Ub conjugates.^12^

In this study, to overcome this limitation, we generated linkage‐ and length‐defined Ub chains in high purity. To do so, we combined our approach to generate triazole‐linked Ub chains of defined linkage type with gel‐eluted liquid fraction entrapment electrophoresis (GELFrEE) fractionation to obtain length‐defined Ub chains.10a

To generate these chains we applied copper(I)‐catalyzed alkyne‐azide cycloaddition (CuAAC),10a, 13 which builds on the pioneering work of Huisgen to generate triazoles by the 1,3‐dipolar cycloaddition between acetylenes and azides.^14^ With sufficient amounts of linkage‐ and length‐defined Ub chains of different polymerization levels (Ub_2_, Ub_4_, and Ub_6+_) in hand, we performed a proteome‐wide screen to identify length‐selective interaction partners for different linkage types (Figure 1). Using HEK293T whole cell lysate, we identified more than 110 significantly enriched interacting proteins for homotypic K27‐, K29‐, and K33‐linked Ub chains, many of which show clear length‐specific binding in addition to their linkage‐specific interaction. These length‐selective interactions identified by proteomic profiling were subsequently confirmed for several proteins by immunoblotting. Thus, our study provides strong evidence that chain length indeed has a significant impact on the recognition of differently linked Ub chains by ubiquitin‐binding proteins (UBPs) and, thus, on the eventual fate of the corresponding modified proteins.

**Figure 1:**
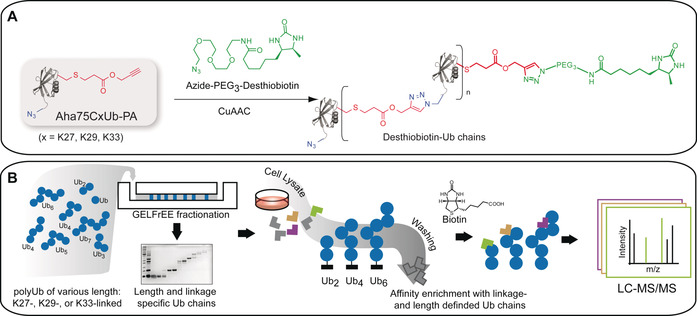
AP‐MS‐based identification of interaction partners of linkage‐ and length‐selective Ub chains. A) Generation of linkage‐specific, desthiobiotin‐modified Ub chains by CuAAC. This one‐pot approach is unique in its ability to produce linkage‐defined Ub chains in high purity and in large enough quantities to enable proteome‐wide studies, and also generates a—normally unmodified—lysine residue in the distal ubiquitin harboring a PA linker and the desthiobiotin affinity tag. B) GELFrEE fractionation of linkage‐defined Ub chains of various polymerization levels generated by CuAAC. Ub_2_, Ub_4_, and Ub_6+_ linked through K27, K29, or K33, were used as the affinity matrix. Enriched proteins were identified by LC‐MS/MS followed by label‐free quantification and statistical analysis.

Analogous to our previous studies,10a, 13a we generated linkage‐defined Ub chains using a bifunctional Ub monomer Aha75CxPA (deletion of G76, replacement of G75 by azidohomoalanine Aha, replacement of K by C at position *x=*27, 29, or 33, and subsequent modification with propargyl acrylate PA). This monomer was used for CuAAC‐mediated protein polymerization and simultaneous modification with desthiobiotin (Figure 1 A). This resulted in the efficient generation of linkage‐defined Ub chains of various polymerization levels. Furthermore, the Ub moieties are linked through triazole linkages, thus making them resistant to DUB‐mediated hydrolysis.10a In this study, we focused on the less‐well characterized homotypic K27‐, K29‐, and K33‐linked Ub chains. After generation of the polymers, they were separated by GELFrEEfractionation (as described in detail in Figure S1 A in the Supporting Information). This enabled the length‐dependent separation of the Ub polymers up to the tetramer level in high purity (Figure S1 B); longer polymers were obtained as mixtures.

As GELFrEEfractionation was performed under denaturing conditions, the separated polymers were refolded by dialysis to restore the native conformation of the polymers (Figure S3). For the investigation of length‐specific interactions, the separated, desthiobiotin‐modified polymers (Ub_2_, Ub_4_, Ub_6+_) of each linkage type (K27, K29, K33) were immobilized on streptavidin agarose. The fraction designated Ub_6+_ contained Ub hexamers and longer polymers.

Having length‐ and linkage‐defined Ub chains in hand, we performed affinity enrichment assays with HEK293T whole cell lysate (Figure S2). Eluted fractions were analyzed by LC‐MS/MS followed by label‐free quantification.^15^ Significantly enriched proteins were identified by ANOVA statistics (FDR=0.02 and S0=2; for a full list see Table S1). Correlation‐based clustering of the 114 significantly enriched interactors was applied to visualize the binding behavior of these proteins to the various Ub chain species. The resulting heat map depicts significantly enriched proteins (Figure 2 A), whereas the Venn diagrams in Figure 2 B provide an overview on the length‐selective binding of the identified proteins. As a consequence of the nature of our Ub chains, we cannot fully exclude the possibility that the Ub dimers may bind additional partners, whose interaction surface may be concealed by the PA linker (see legend to Figure 1). For our analysis we have, thus, refrained from putting special emphasis on Ub dimers but rather consider them in combination with their Ub_4_ counterparts—that is, as shorter Ub polymers (Ub_2_, Ub_4_) versus longer polymers (Ub_6_ and higher). In the proteomic data from our affinity enrichment assays, we detected clear length‐dependent binding of many proteins in addition to the linkage‐selective binding.

**Figure 2:**
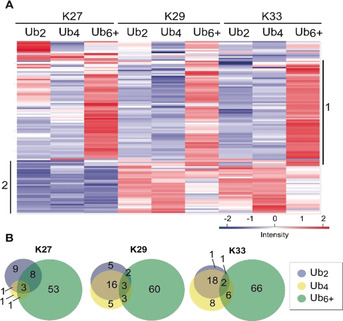
Statistical analysis of affinity enrichment assays with linkage‐ and length‐defined Ub chains. A) Hierarchical clustering of statistically significant interactions. The interacting proteins are shown in rows; columns depict the Ub variant used as the affinity matrix. Numbers on the *y*‐axis indicate clusters with similar interaction behavior of Ub chain binders. Cluster 1: proteins interacting preferentially with long chains (Ub_6+_); cluster 2: proteins showing a similar interaction pattern with K29‐ and K33‐linked Ub chains. B) Overview of length‐selective interactions.

For longer polymers (Ub_6+_), in particular, we observed large clusters of proteins that were significantly more enriched than shorter polymers (Ub_2_, Ub_4_) of the same linkage type (heat map cluster 1 in Figure 2 A; Figure 2 B). For each of the herein‐studied three linkage types (K27, K29, K33), 64 % to 70 % of the significant interactions were found exclusively with long polymers (Ub_6+_; Figure 2 B). It has been shown that Ub chains of a certain linkage type do not only adopt a single conformation but rather an ensemble of conformations specific to the linkage type.^16^ Thus, it seems likely that longer polymers can adopt a higher number of different conformations than dimers, which would allow longer polymers to provide more orientations of specific interaction sites for Ub binders.

Besides proteins with a preference for longer Ub chains, our analysis also revealed proteins that appear to interact preferentially with shorter chains (Ub_2_, Ub_4_; cluster 2, Figure 2 A) Here, the similarity of K29‐ and K33‐linked Ub_2_ and Ub_4_ chains with respect to their respective interaction partners is striking (cluster 2 in Figure 2 A). We speculate that this selectivity might arise from unique conformations of short polymers that cannot be adopted by longer polymers and, thus, are preferentially recognized by certain UBPs. Furthermore, in an earlier study10a we already observed a significant overlap of proteins binding to K29‐ and K33‐linked chains, thus suggesting these two linkage types have similar biological functions. A similar behavior is also indicated by structural simulations and NMR studies of all seven K‐linked Ub dimers, which show the highest similarities between K29‐ and K33‐linked dimers compared to all other linkage types.^16^

To verify the chain‐length‐specific interactions observed by LC‐MS/MS, we selected several proteins and also studied their binding behavior by affinity enrichment followed by Western blot analysis. The first interaction partner analyzed was Ubac1 (the noncatalytic subunit of the E3 ligase complex KPC), which is involved in poly‐ubiquitylation and proteasomal degradation of CDKN1B during the cell cycle.^17^ Our data illustrated that Ubac1 (located in cluster 2, Figure 2 A) showed preferred binding to shorter Ub chains with a preference for Ub_2_/Ub_4_ over Ub_6+_ for K29‐ and K33‐linked Ub chains. Indeed, the Western blot analysis confirmed not only the preferential interaction of Ubac1 with Ub_4_ (shown exemplarily for K33‐linked Ub chains) but also the linkage selectivity (Figure 3 A, top left; Figure 3 B). We also studied the catalytic subunit of the E3 ligase complex KPC, RNF123 (located in cluster 2; Figure 2 A), which showed similar enrichment patterns as Ubac1, with a preference for shorter Ub chains and the strongest binding to K29‐ and K33‐linked Ub_4_, but no apparent binding to K27‐linked polymers. Again, Western blot analysis verified the linkage‐ and length‐selective binding of RNF123 to K33‐linked Ub_4_, which is in excellent agreement with our AP‐MS analysis (Figure 3 A, bottom left; Figure 3 B). As Ubac1 and RNF123 show identical binding behavior to the Ub chain species, it is tempting to speculate that the whole KPC complex is bound by these Ub chains. Since we used whole cell extracts for our analysis, more detailed binding studies will be necessary to determine which of the KPC subunits is responsible for the selective binding of Ub chains. We also confirmed the selective interaction of the DUB USP15, especially with long K29‐ and K33‐linked polymers (Ub_6+_). USP15 is involved in the regulation of various pathways, such as NF‐kB signaling and mitophagy, where it acts as an inhibitor by counteracting the action of RNF41‐PRKN.^18^ Again, the results of the Western blot analysis were in excellent agreement with the MS results (Figure 3 A, right; Figure 3 B). Taken together, our immunoblotting data strongly confirmed the linkage‐ and length‐specific interactions from our AP‐MS data, thus validating our proteomic profiling approach. How Ub chain length specificity is realized by Ubac1, RNF123, and USP15 remains unclear. We speculate that characteristic positioning of UBDs in these proteins mediates specific recognition of a certain chain length, potentially by offering additional interaction surface.

**Figure 3:**
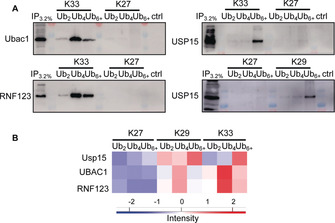
Validation of length‐selective interactions by immunoblotting. A) Validation of interactions by Western blot analysis. Elution fractions of the affinity enrichment were subjected to Western blot analysis with antibodies specific for the protein indicated. IP=Input. B) Heatmap indicating enrichment of selected proteins according to the MS measurement.

In this study, we investigated the impact of chain length on the interactome of differently linked Ub chains on a proteome‐wide scale. To do so, we have critically expanded a procedure for the generation of linkage‐defined, nonhydrolyzable Ub chains10a, 13a by including a high‐resolution separation step of the Ub chains. The resulting linkage‐ and length‐defined Ub chains were employed in AP‐MS‐based proteomic profiling, thereby enabling the identification of a number of proteins that bind to Ub chains in a linkage‐ and length‐selective manner. Since selected interactions were confirmed by immunoblot analysis, we conclude that in addition to already known features such as linkage type, branching level, or modification of Ub itself (e.g. phosphorylation and acetylation), the length of the Ub chains adds another layer of complexity to the Ub code. Up to now, it is not known how the Ub chain length may affect the interaction with UBPs and thus Ub signaling, as there is only limited information on the mode of interaction of UBPs with long Ub chains in general^19^ and the potential preference for a certain chain length in particular.^8^, ^20^

Interestingly, we find that K29‐ and K33‐linked hexamers and longer chains seem to exhibit an increased tendency to bind metabolite interconversion enzymes [e.g. hydrolases, transferases, or oxidoreductases (Figure S4)], while shorter K29‐ and K33‐linked chains (Ub_2_, Ub_4_) show a tendency to interact less with this protein class but bind preferentially protein‐modifying enzymes [e.g. kinases, proteases, or Ub ligases) (Figure S4)]. This is a first indication that Ub chain length may also implicate functional consequences with regard to their respective UBPs. In this respect future detailed investigations will be required to fully dissect how different chain lengths are recognized by different UBPs.

## Conflict of interest

The authors declare no conflict of interest.

## Supporting information

As a service to our authors and readers, this journal provides supporting information supplied by the authors. Such materials are peer reviewed and may be re‐organized for online delivery, but are not copy‐edited or typeset. Technical support issues arising from supporting information (other than missing files) should be addressed to the authors.

Supplementary
