# Supplementary material for: The Length of a Ubiquitin Chain: A General Factor for Selective Recognition by Ubiquitin‐Binding Proteins
Source: Angew Chem Int Ed Engl. 2020 Jun 8;59(30):12371–5. doi: 10.1002/anie.202003058 (PMC7384046; doi:10.1002/anie.202003058)
Supplement: Supplementary file 1 — Supplementary [file ANIE-59-12371-s001.pdf]

Angewandte Chemie
Eine Zeitschrift der Gesellschaft Deutscher Chemiker

Supporting Information

The Length of a Ubiquitin Chain: A General Factor for Selective Recognition by Ubiquitin-Binding Proteins

Joachim Lutz[+], Eva Höllmüller[+], Martin Scheffner, Andreas Marx,* and Florian Stengel**

anie_202003058_sm_miscellaneous_information.pdf

Experimental Procedures

Expression and purification of Aha75CxUb (x = 27, 29 or 33)

The Ub variants were generated as described [1]. In short, the cDNA encoding human G75MUb (G76 deleted) was cloned into the pGEX2TK vector by BamH I / EcoR I double digest and T4 DNA ligase (NEB) ligation. To construct the pGEX2TK-TEV-G75MKxCUb plasmids, site-directed mutagenesis was performed to replace a single lysine codon at position x (x = 27, 29, or 33) by a cysteine codon.

Methionine (Met) auxotrophic E.coli B834 (DE3) transformed with pGEX2TK-TEV-G75MKxCUb was cultured in LB medium containing 100 mg/L carbenicillin at 37 °C overnight. The pre-culture was diluted with NMM medium containing 0.04 mM Met and 100 mg/L carbenicillin to an OD600 value of 0.1. Cells were grown at 37 °C until they reached an OD600 of approximately 1.3, harvested and resuspended in the same volume of fresh NMM supplemented with 0.5 mM Aha. After incubation at 37 °C for 30 min, expression of GST-fusion proteins was induced by addition of 1 mM IPTG and cells were further incubated overnight at 25 °C. Then, cells were pelleted, resuspended in 1xPBS buffer containing 1% Triton X-100, and lysed by sonication. The lysate was clarified by high speed centrifugation and the supernatant was incubated with glutathione agarose beads at 4 °C for 5 h. The beads were transferred into a column, washed three times with 1xPBS, and incubated with 0.33 mg of TEV protease at room temperature overnight. The released Aha75CxUbs were eluted with (10x0.3 ml) of 1xPBS. Protein purity was analyzed by 15% SDS-PAGE followed by Coomassie blue staining, and the concentration was measured through BCA protein assay (ThermoFisher).

Modification of Aha75CxUb by propargyl acrylate (PA) as published in [1]

10 µM of Aha75CxUb were incubated with 100 eq. propargyl acrylate (PA) in 20 mM Tris-HCl (pH 8.0)/MeCN= 9:1 at 37 °C by shaking at 180 rpm for 2 h. 20 µL of reaction mixtures were withdrawn and reacted with 25 eq. fluorescein-5-maleimide (F5M) in the dark at 37 °C for 20 min. As control, the same amount of PA-untreated Aha75CxUb was incubated with F5M under the same conditions. All the samples were resolved by SDS-PAGE and visualized under UV-light. The same gel was subsequently stained by Coomassie blue. To remove excess PA, reaction mixtures were transferred to dialysis tubes (3500 MWCO) and dialyzed sequentially against 20 mM Tris-HCl (pH 7.5)/MeOH= 4:1, and 20 mM Tris-HCl (pH 7.5).

Generation of linkage and length defined Ub chains

Generation of desthiobiotin-modified Ub chains was performed by CuAAC in 20 mM Tris-HCl (pH 8) supplemented with 0.5 mM SDS under argon atmosphere. Aha75C27Ub-PA (120 µM) was mixed with 5 mM THPTA (Tris(3-hydroxypropyltriazolylmethyl)amine) before the reaction mixture was flushed with argon. The reaction was initiated by adding 2.5 mM Cu(MeCN)4BF4. After 5 min, 2.5 mM azide-PEG3-desthiobiotin (Jena Bioscience) were added and the reaction mixture was flushed with argon. After incubation on ice for 1 h, the reaction was quenched with 15 mM EDTA and dialyzed at 4 °C overnight against 1xPBS (pH 7.4) with 200-fold buffer excess [1].

Separation of Ub chains according to their length

While our "one-pot" approach is able to generate all Ub polymers up to long polymerization levels (Ub_2, Ub_3, Ub_4…), we decided to concentrate on dimers, tetramers and longer polymers (Ub_{6+}) for our investigation, as they could be clearly separated from each other (e.g. trimers overlapped with both a fraction of dimers and a fraction of tetramers) and have thus chosen solely those polymers after separation via the Gel Eluted Liquid Fraction Entrapment Electrophoresis (GELFrEE) technology for further processing. Ub chains were desalted by dialysis before the samples were concentrated by a SpeedVac vacuum concentrator followed by determination of the protein concentration by BCA assay. Samples were prepared for GELFrEE fractionation according to the manufacturer`s instructions (Expedeon). In brief, 0.4 mg of desalted Ub chains were mixed with 30 µl acetate sample buffer (5x), 8 µl 1 M DTT and MilliQ in order to adjust the sample volume to 150 µl. The sample was heated to 56 °C for several minutes before loading on the 10% Tris-Acetate Cartridge. During the separation, samples were taken at defined times according to the manufacturer's protocol. Purity of samples was analyzed by SDS-PAGE followed by Coomassie blue staining. Fractions containing the desired Ub chains were first dialyzed for 48 h

to MilliQ in order to remove SDS before the samples were supplemented with 8 M Urea in 20 mM Tris-HCl pH 7.5. Refolding of the protein was then accomplished by dialysis to 20 mM Tris-HCl pH 7.5, 50 mM NaCl. Structural integrity was confirmed by CD analysis (Supplementary Fig. 2). Comparative analysis of different Ub chain linkage types resulted in very similar spectra, due to identical secondary structure elements in all three chain types (data not shown). Samples were concentrated using Amicon centrifugal units with 10 kDa molecular mass cut-off, before the concentration was measured again by BCA assay and purity was analyzed by SDS-PAGE analysis followed by Coomassie blue staining. For proof of the correct linkage type for the various Ub chains generated by CuAAC by LC-MS/MS analysis please see our previous work [1].

Preparation of HEK293T cell lysate

Cells were grown in DMEM supplemented with 10% (v/v) FBS at 37 °C, 5% -vol CO2. For lysis, cells were centrifuged at 500 x g for 10 min. Cell pellets were washed with ice-cold 1x PBS, resuspended in ice-cold lysis buffer (25 mM Tris-HCl, pH 7.5, 150 mM NaCl, 1 mM DTT, 1 mM EDTA, 1 % NP40, 0.1 M Pefabloc, 1 µg/mL Aprotinin/Leupeptin) and incubated on ice for 20 min. Lysates were centrifuged at 14000 x g for 20 min. The supernatant was stored on ice until further use.

Affinity enrichment assay with Ub chains (exemplary for K29-linked chains)

20 µg length-separated desthiobiotin-labeled K29-linked Ub chains (Ub_2, Ub_4, Ub_{6+}) were incubated for 3 h at 4 °C with streptavidin agarose beads (0.7 µg of protein/µl of bead slurry). After washing the beads three times with buffer (25 mM Tris-HCl, pH 7.5, 500 mM NaCl, 1 mM DTT, 1 mM EDTA, 1 % NP-40), HEK293T cell lysate (3.5 mg/mL) was added. The mixture was incubated for 3 h at 4 °C. The beads were pelleted by centrifugation (500 x g) and washed seven times with lysis buffer (25 mM Tris-HCl, pH 7.5, 150 mM NaCl, 1 mM DTT, 1 mM EDTA, 1 % NP40, Pefablock 0.1 M, Aprotinin/Leupeptin 1 µg/mL). Elution of bound proteins was performed with 0.8 mM biotin in 20 mM Tris-HCl (pH 7.5) at 37 °C. Elution fractions were collected and analyzed by SDS-PAGE.

In-gel digestion as published in [1]

For in-gel digestion a slightly modified protocol adapted from [2] was used. Briefly, elution fractions of the respective enrichment were separated by SDS-PAGE followed by Coomassie staining. Each gel lane was cut into 4 pieces and destained in 50 mM NH4HCO3/MeCN (1:1, v/v). After washing with 50 mM NH4HCO3, proteins were reduced by the addition of 10 mM DTT in 50 mM NH4HCO3 for 60 min at 56 °C followed by alkylation in 50 mM iodoacetamide in 50 mM NH4HCO3 for 60 min at room temperature. After washing in 50 mM NH4HCO3/MeCN (1:1, v/v) and dehydration in MeCN, proteins were digested overnight at 37 °C with trypsin (1:50, w/w) (Promega V5111). Peptides were extracted from the gel in 5 % MeCN/0.1 % formic acid and desalted using U-C18 ZipTips (Merck Millipore).

Mass spectrometry

Tryptic peptides were separated on an EASY-nLC 1200 system (Thermo Scientific) at a flow rate of 300 nL/min using a 45 min gradient from 5 % ACN/0.1 % formic acid to 35 % ACN/0.1 % formic acid and 5 min to 45 % ACN/0.1 % formic acid followed by a washing step at 80 % ACN. Mass spectra were recorded on a Q Exactive HF mass spectrometer (Thermo Scientific) operated in data dependent Top20 mode with dynamic exclusion set to 40 s. Full scan MS spectra were acquired at a resolution of 120,000 (at m/z 200) with an automatic gain control target value of $3e^6$ and a maximum injection time of 60 ms. Most intense precursors with charge states of 2-6 reaching a minimum automatic gain control target value of $2e^3$ were selected for MS/MS experiments. Normalized collision energy was set to 28. MS/MS spectra were collected at a resolution of 15000 (at m/z 200), an automatic gain control target value of $1e^5$ and 100 ms maximum injection time. Each of the biological triplicates was measured as a technical duplicate.

Mass spectrometry data analysis and quantification

Raw files from LC-MS/MS measurements were analyzed using MaxQuant (version 1.6.1.0) [3] with match between runs and label-free quantification (LFQ) (minimum ratio count 2) enabled. The minimal peptide length was set to 5. For protein identification, the human reference proteome downloaded from the UniProt database (download date: 2018-02-22) and the integrated database of common contaminants were used.

Further data processing was performed using Perseus software (version 1.6.1.3) [4]. Identified proteins were filtered for reverse hits, common contaminants and proteins that were only identified by site. LFQ intensities were log2 transformed, filtered to be detected in at least 4 out of 6 replicates and missing values were imputed from a normal distribution (width = 0.3 and shift = 1.8), based on the assumption that these proteins were below the detection limit. Significantly enriched proteins were identified by an ANOVA test (FDR = 0.02, s0 = 2), averaged and normalized by Z-scoring. Finally, the enriched proteins were analyzed by hierarchical clustering (Euclidean distance) and plotted as heatmap. Proteins with a minimum Z-score of 0.5 are indicated as enriched in the respective sample (Supplementary Table 1). Venn diagrams were generated using BioVenn [5].

Immunoblotting

For verification of interactions by immunoblotting, the amount of loaded protein was scaled down by a factor of 4 compared to the MS/MS analysis of the affinity enrichment assays. Input represents 3.2 % of the HEK293T cell lysate used for affinity enrichment. Used antibodies were directed against RNF123 (sc-101122, Santa Cruz Biotechnology), UBAC1 (ab151317, abcam), USP15 (ab71713, abcam).

Data availability

All data generated or analyzed during this study are included in this published article (and its supplementary information files). The MS raw files have been deposited to the ProteomeXchange Consortium via the PRIDE [6] partner repository with the project accession number: PXD015877 (Username: reviewer62730@ebi.ac.uk; Password: Rz6W2EwA)

Supplementary Fig. 1

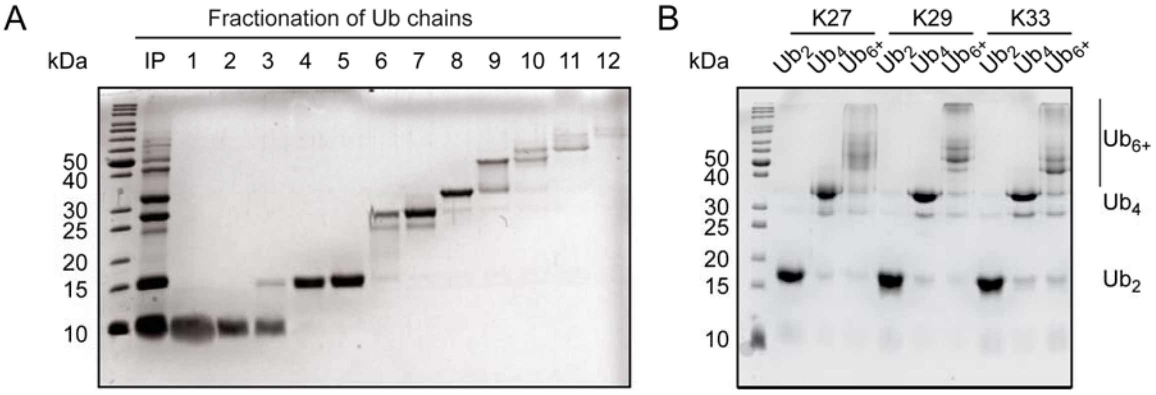

Supplementary Fig 1 GELFrEE fractionation system. (A) SDS-PAGE analysis of a GELFrEE fractionation run with Ub chains. Input (IP) shows the Ub chains before fractionation. Numbers indicate elution fractions collected during the fractionation. **(B)** SDS-PAGE of linkage- and length-defined Ub chains generated by GELFrEE fractionation. Ub chains were visualized by Coomassie blue staining.

Supplementary Fig. 2

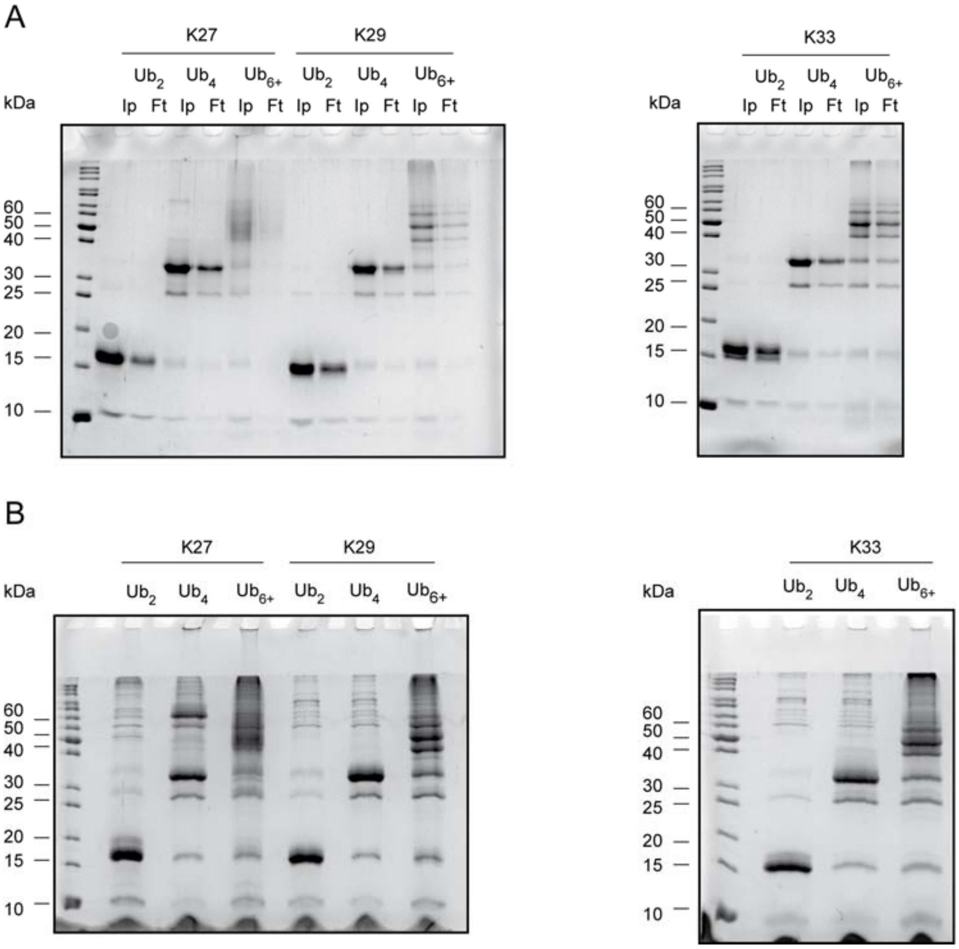

Supplementary Fig. 2 Analysis of the affinity purification procedure for the identification of length-selective Ub binding proteins using desthiobiotin-labelled Ub chains of defined length (Ub_2, Ub_4 Ub_{6+}) as matrix. (A) The respective Ub species (Input, Ip) were incubated with Streptavidin agarose. Subsequently, the flow through (Ft) was collected and agarose beads were washed. **(B)** Bound proteins were eluted and separated by SDS-PAGE followed by Coomassie Blue staining. Each lane was excised and cut into four pieces prior to LC MS/MS analysis.

Supplementary Fig. 3

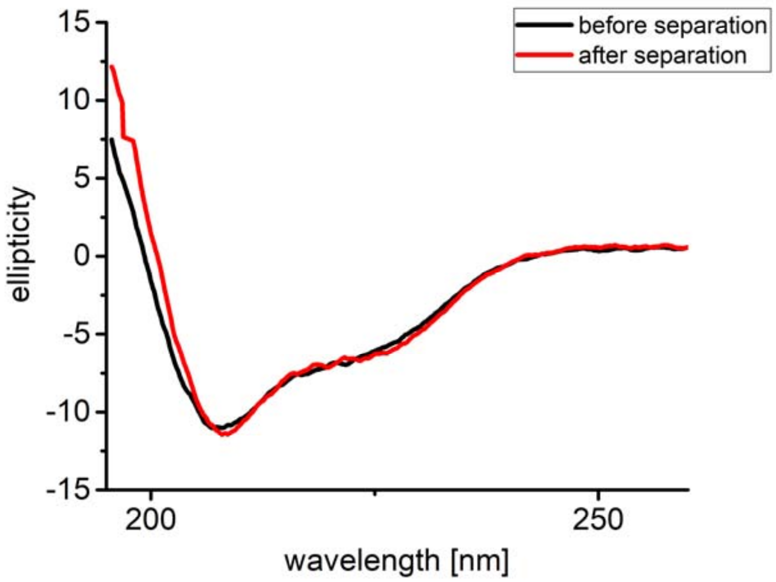

Supplementary Fig. 3, Secondary structure analysis of Ub chains before and after GELFrEE **fractionation.** Secondary structure analysis of K33-linked Ub chains. (Black) Ub chains (all polymerization levels) before GELFrEE fractionation, (Red) Ub chains after GELFrEE fractionation and refolding (after separation, separated polymers were pooled again). All samples were measured at 0.2 mg/ml concentration in 5 mM Tris-HCl pH 7.5.

Supplementary Fig. 4

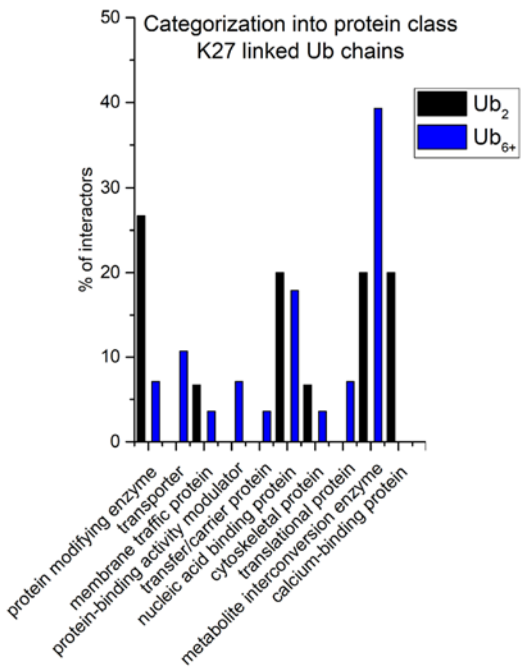

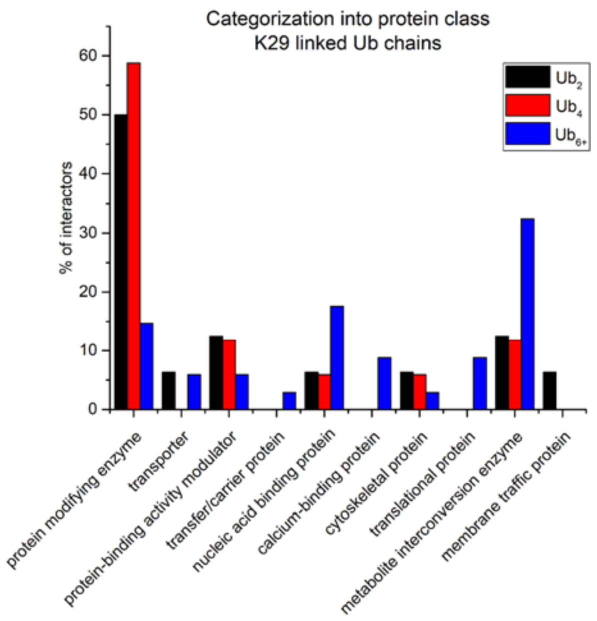

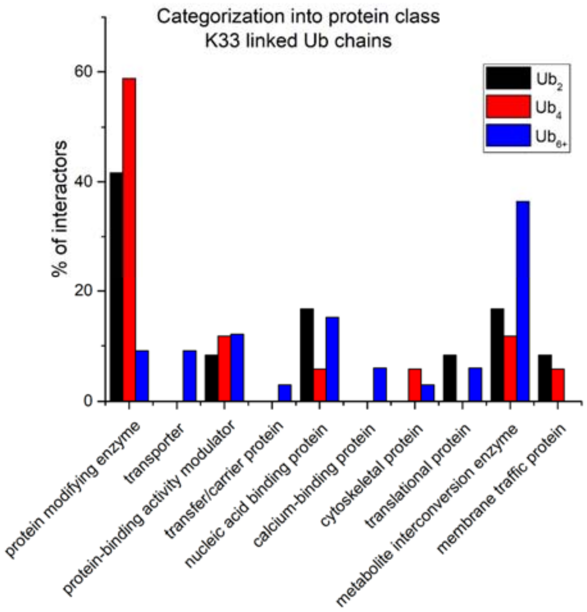

Supplementary Fig 4 GO-term analysis based on PANTHER classification [7]. Proteins significantly enriched in ANOVA analysis were classified according to protein classes. Significantly enriched proteins with respect to linkage type and polymer length (Ub_2, Ub_4 Ub_{6+}) were analyzed. Values represent the proportion of interaction partners assigned to a certain protein class with respect to the total number of protein class hits. For the group of K27 linked Ub_4 selective binders, the classification was omitted due to a too low number of enriched proteins.

References

[1] X. Zhao, J. Lutz, E. Höllmüller, M. Scheffner, A. Marx, F. Stengel, *Angew. Chem. Int. Ed.* **2017**, *56*, 15764-15768.

[2] A. Shevchenko, H. Tomas, J. Havli, J. V. Olsen, M. Mann, *Nat. Protoc.* **2006**, *1*, 2856-2860.

[3] a) J. Cox, M. Y. Hein, C. A. Luber, I. Paron, N. Nagaraj, M. Mann, *Mol. Cell. Proteomics* **2014**, *13*, 2513-2526. b) J. Cox, M. Mann, *Nat. Biotech.* **2008**, *26*, 1367-1372.

[4] S. Tyanova, T. Temu, P. Sinitcyn, A. Carlson, M. Y. Hein, T. Geiger, M. Mann, J. Cox, *Nat. Meth.* **2016**, *13*, 731-740.

[5] T. Hulsen, J. de Vlieg, W. Alkema, *BMC Genom.* **2008**, *9,* 488.

[6] Y. Perez-Riverol, A. Csordas, J. Bai, M. Bernal-Llinares, S. Hewapathirana, *Nucleic Acids Res* **2019**, *47*, D442-D450.

[7] H. Mi, A. Muruganujan, D. Ebert, X. Huang, P. D. Thomas, *Nucleic Acids Res* **2019**, *47*, D419-D426.

Enriched proteins - Gene names

K27_Dimer	K27_Tetramer	K27_Poly	K27_all	K29_Dimer	K29_Tetramer	K29_Poly	K29_all	K33_Dimer	K33_Tetramer	K33_Poly	K33_all
ZUFSP	TIPRL	NDUFS2	ZUFSP	TCAF1	WDTC1	RNF31	TCAF1	PCNA	UBAC2	UBAC1	PCNA
PPT1	WDTC1	RUVBL1	PPT1	HERC2	HECTD1	PEX16	HERC2	UBAC2	TBC1D15	SDF4	UBAC2
ERAL1	PLAA	NUP214	ERAL1	USP15	TBC1D15	WRNIP1	USP15	TBC1D15	UBR5	RCN1	TBC1D15
GCDH	SORT1	WDTC1	GCDH	CUEDC1	ZRANB1	WDTC1	CUEDC1	DDX41	HECTD1	RNF123	DDX41
RNASEH2A	RPL14	PPT1	RNASEH2A	TOM1L2	MIB2	TBC1D15	TOM1L2	WRNIP1	TCAF1	HERC2	WRNIP1
NTPCR	CPVL	GCDH	NTPCR	HECTD1	PRKCI	UBAC2	HECTD1	TOM1	UCHL1	NUP214	TOM1
SDF4		TUBB4A	SDF4	SQSTM1	WRNIP1	TUBB4A	SQSTM1	LLGL1	WRNIP1	NDUFA4	LLGL1
RCN1		RNASEH2A	RCN1	TBC1D15	UBAC1	RER1	TBC1D15	TOM1L2	ZUFSP	TBC1D15	TOM1L2
LANCL1		MLEC	LANCL1	HADHB	TCAF1	SDF2L1	HADHB	SQSTM1	USP5	PCNA	SQSTM1
TBK1		CDK1;CDC2	TBK1	TAX1BP1	RNF123	SLC25A1	TAX1BP1	USP5	ZRANB1	DERL1	USP5
TUBB4A		PFKM	TUBB4A	WRNIP1	HADHB	NDUFA4	WRNIP1	USP13	PRKCI	TCAF1	USP13
NDUFS2		RALA	NDUFS2	HADHA	USP13	PTPMT1	HADHA	RPL14	DDX41	TBK1	RPL14
PCNA		DNAJA3	PCNA	PRKCI	SQSTM1	PAM16;CORO7-PAM16	PRKCI	TAX1BP1	USP13	RNASEH2A	TAX1BP1
WDTC1		FAF2	WDTC1	OTUB1	TMEM160	PET100	OTUB1	HADHB	TAX1BP1	SLC25A11	HADHB
PLAA		ERAL1	PLAA	NUP214	TAX1BP1	AGK	NUP214	HECTD1	SQSTM1	NTPCR	HECTD1
SORT1		APOL2	SORT1	NEURL4	USP5	QPCTL	NEURL4	HADHA	LLGL1	TMEM33	HADHA
CPVL		HMOX1	CPVL	USP13	LLGL1	TCAF1	USP13	GBAS	HADHA	TOMM5	GBAS
DNAJB11		PET100	DNAJB11	TMEM160	SHARPIN	HNRNPUL1	TMEM160	NIPSNAP1	HERC2	DHCR24;Nbla03646	NIPSNAP1
CALU		RAB9A	CALU	NIPSNAP1	HERC2	TMEM126A	NIPSNAP1	EPDR1	GBAS	ERAL1	EPDR1
SDF2		ATP5C1	SDF2	LLGL1	TOM1	TECR	LLGL1	PRKCI	TOM1L2	TECR	PRKCI
SDF2L1		PLAA	SDF2L1	MYO6	TOM1L2	DNAJA3	MYO6	UCHL1	OTUB1	PFKM	UCHL1
		LANCL1	TIPRL	USP5	HADHA	SDF4	USP5	OTUB1	MYO6	TUBB4A	OTUB1
		RPS5	RPL14	EPDR1	RNF31	RCN1	EPDR1		EPDR1	HMOX1	UBR5
		NTPCR	RUVBL1	ZRANB1	NEURL4	TMEM33	ZRANB1		HADHB	CDK1;CDC2	TCAF1
		HNRNPUL1	NUP214	GBAS	RBCK1	HSD17B12	GBAS		RNF31	APOL2	ZUFSP
		TFB2M	MLEC	UCHL1	CUEDC1	DERL1	UCHL1		NIPSNAP1	ATP5C1	ZRANB1
		VWA8	CDK1;CDC2		MYO6	ZUFSP	WDTC1		NEURL4	MLEC	HERC2
		IRS4	PFKM			CALU	MIB2		SHARPIN	NDUFS2	MYO6
		TOMM5	RALA			RPS5	UBAC1		TMEM160	PPT1	RNF31
		DNAJB6	DNAJA3			FARSA	RNF123		RBCK1	SEC22B	NEURL4
		RAB35	FAF2			MGAT2	SHARPIN		TOM1	RAB9A	SHARPIN
		AGK	APOL2			RPL14	TOM1		CUEDC1	QPCTL	TMEM160
		MGAT2	HMOX1			ARMCX3	RNF31		RNF123	TAF6L	RBCK1
		DPM1	PET100			CDK1;CDC2	RBCK1		UBAC1	GCDH	CUEDC1
		NDUFA9	RAB9A			IRS4	PEX16			VWA8	RNF123
		SORT1	ATP5C1			DPM1	UBAC2			DPM1	UBAC1
		PAM16;CORO7-PAM16	RPS5			DNAJB6	TUBB4A			TMEM126A	SDF4
		ARMCX3	HNRNPUL1			NDUFA9	RER1			RPS5	RCN1
		TAF6L	TFB2M			LANCL1	SDF2L1			UBAC2	NUP214
		SEC22B	VWA8			NTPCR	SLC25A1			UFSP2	NDUFA4
		RAB8A	IRS4			PFKM	NDUFA4			DNAJB6	DERL1
		PTPMT1	TOMM5			ATP5C1	PTPMT1			SLC25A1	TBK1
		FARSA	DNAJB6			ERAL1	PAM16;CORO7-PAM16			HSD17B12	RNASEH2A
		HSD17B12	RAB35			RALA	PET100			TFB2M	SLC25A11
		SLC25A11	AGK			RAB35	AGK			FAF2	NTPCR
		SLC25A1	MGAT2			SEC22B	QPCTL			IRS4	TMEM33
		TMEM33	DPM1			TFB2M	HNRNPUL1			FARSA	TOMM5
		COX6C	NDUFA9			UBR5	TMEM126A			RAB8A	DHCR24;Nbla03646
		DNAJC19	PAM16;CORO7-PAM16			TBK1	TECR			SLC27A4	ERAL1
		TAMM41	ARMCX3			RAB8A	DNAJA3			RER1	TECR
		PEX16	TAF6L			PPT1	SDF4			USP15	PFKM
		PHKB	SEC22B			HMOX1	RCN1			UBR5	TUBB4A
		RER1	RAB8A			MIB2	TMEM33			FAR1	HMOX1
		POLE4	PTPMT1			GCDH	HSD17B12			DNAJA3	CDK1;CDC2
		TMEM126A	FARSA			TOMM5	DERL1			NDUFA9	APOL2
		TECR	HSD17B12			TAF6L	ZUFSP			DHRS7B	ATP5C1
		FAR1	SLC25A11			FAF2	CALU			RAB35	MLEC
		NCBP2-AS2	SLC25A1			APOL2	RPS5			ARMCX3	NDUFS2
		DERL1	TMEM33			NUP214	FARSA			PEX16	PPT1
		QPCTL	COX6C			USP15	MGAT2			NCBP2-AS2	SEC22B
		DHRS7B	DNAJC19			VWA8	RPL14			MIB2	RAB9A
		SLC27A4	TAMM41			MLEC	ARMCX3			AGK	QPCTL
		NDUFA4	PEX16			DDX41	CDK1;CDC2			ADPGK	TAF6L
		DHCR24;Nbla03646	PHKB			RNASEH2A	IRS4			COX6C	GCDH
		TIPRL	RER1			POLE4	DPM1			ZUFSP	VWA8
			POLE4			RAB9A	DNAJB6			PAM16;CORO7-PAM16	DPM1
			TMEM126A			ADPGK	NDUFA9			MGAT2	TMEM126A
			TECR			UFSP2	LANCL1			TAMM41	RPS5
			FAR1				NTPCR			PTPMT1	UFSP2
			NCBP2-AS2				PFKM			RALA	DNAJB6
			DERL1				ATP5C1			PHKB	SLC25A1
			QPCTL				ERAL1			RUVBL1	HSD17B12
			DHRS7B				RALA			DNAJC19	TFB2M
			SLC27A4				RAB35			PET100	FAF2
			NDUFA4				SEC22B			RUVBL2	IRS4
			DHCR24;Nbla03646				TFB2M				FARSA
							UBR5				RAB8A
							TBK1				SLC27A4
							RAB8A				RER1
							PPT1				USP15
							HMOX1				FAR1
							GCDH				DNAJA3
							TOMM5				NDUFA9
							TAF6L				DHRS7B
							FAF2				RAB35
							APOL2				ARMCX3
							VWA8				PEX16
							MLEC				NCBP2-AS2
							DDX41				MIB2
							RNASEH2A				AGK
							POLE4				ADPGK
							RAB9A				COX6C
							ADPGK				PAM16;CORO7-PAM16
							UFSP2				MGAT2
											TAMM41
											PTPMT1
											RALA
											PHKB
											RUVBL1
											DNAJC19
											PET100
											RUVBL2

		Length + linkage selectivity									Average enrichment, Z-scored (log2)								
Protein names	Gene names	K27 Dimer	K27 Tetramer	K27 Poly	K29 Dimer	K29 Tetramer	K29 Poly	K33 Dimer	K33 Tetramer	K33 Poly	K27 Dimer	K27 Tetramer	K27 Poly	K29 Dimer	K29 Tetramer	K29 Poly	K33 Dimer	K33 Tetramer	K33 Poly
Sortilin	SORT1	++	+	++							1.3311	0.969211	1.30685	-0.657848	-1.32622	0.0173265	-0.83318	-0.833146	0.025902
Neuralized-like protein 4	NEURL4				+	++			++		-1.15307	-1.14968	-0.911523	0.995431	1.20349	0.00768013	-0.593483	1.27196	0.329191
E3 ubiquitin-protein ligase HERC2	HERC2				+	++			+	+	-1.11437	-1.69268	-0.943917	0.667608	1.06155	0.140611	0.283939	0.96211	0.635146
ADP-dependent glucokinase	ADPGK						++			++	-0.603435	-0.635559	0.135274	-0.727941	-0.320121	1.71833	-0.682169	-0.56934	1.68496
Mitochondrial import receptor subunit TOM5 homolog	TOMM5			++			++			++	0.407158	-0.703275	1.26813	-1.01348	-0.950937	1.2718	-0.797417	-0.581385	1.09941
von Willebrand factor A domain-containing protein 8	VWA8			++			++			++	-0.576966	-0.499017	1.2519	-0.461552	-0.578775	1.40107	-0.877174	-0.950551	1.29106
Delta(24)-sterol reductase	DHCR24;Nbla03646			+++						++	-0.0831222	-0.838858	2.13656	-0.605513	-0.184224	-0.250288	-0.9789	-0.297672	1.10202
Probable serine carboxypeptidase CPVL	CPVL	++	++								1.38852	1.93162	0.0690675	-0.299843	-0.703922	-0.255319	-0.392637	-0.973348	-0.764141
TAF6-like RNA polymerase II p300/CBP-associated factor-associated factor 65 kDa subunit 6L	TAF6L			++			++			++	-0.389672	-0.461888	1.33229	-0.427803	-0.605296	1.27408	-0.806379	-1.18919	1.27386
Protein PET100 homolog, mitochondrial	PET100			++			+			++	-0.792903	-0.913592	1.09123	-0.369514	-0.362178	0.778269	-0.530243	-0.789839	1.88877
Derlin-1	DERL1			++			+			+	0.0189826	0.136787	1.78086	-0.755631	-1.08767	0.877675	-1.07551	-0.697062	0.801571
Phosphatidate cytidylyltransferase, mitochondrial	TAMM41			++						++	-0.709677	-0.819756	1.62554	-0.491508	-0.500642	0.351958	-0.623813	-0.532909	1.70081
Mammalian ependymin-related protein 1	EPDR1				++			++	++		-0.775872	-0.971746	-1.44455	1.12257	0.409874	-0.378804	1.2265	1.09933	-0.287303
Glutaryl-CoA dehydrogenase, mitochondrial	GCDH	+		+			++			++	0.811799	-0.762816	0.771512	-0.802419	-1.00273	1.26974	-0.891801	-0.677178	1.2839
Heterogeneous nuclear ribonucleoprotein U-like protein 1	HNRNPUL1			++			+				-0.0065183	0.017909	1.2507	0.142118	0.0503136	0.80765	-2.39306	-0.0465269	0.177416
Protein kinase C iota type	PRKCI				+	+		++	+		-1.12943	-1.37984	-1.06138	0.898229	0.795761	-0.0182481	1.23451	0.835894	-0.175506
DnaJ homolog subfamily A member 3, mitochondrial	DNAJA3			++			+			++	0.377701	-1.23486	1.02094	-0.366454	-1.25131	0.830449	-0.551647	-0.388515	1.5637
Cytochrome c oxidase subunit NDUFA4	NDUFA4			+++			+			+	-0.557782	-0.0685009	2.1346	-0.961876	-0.734544	0.705294	-0.628364	-0.56266	0.673836
E3 ubiquitin-protein ligase MIB2	MIB2					+	++			++	-0.891328	-0.825394	-0.479493	-0.632563	0.780774	1.26319	-1.00642	0.163562	1.62767
CUE domain-containing protein 1	CUEDC1				+	++			++		-0.781502	-0.889126	-0.779266	0.684153	1.35033	-0.747659	0.232794	1.61712	-0.68684
Lethal(2) giant larvae protein homolog 1	LLGL1				++	++		+	+		-1.49958	-1.03686	-1.0137	1.04328	1.0359	-0.185077	0.779665	0.918247	-0.0418665
Very-long-chain enoyl-CoA reductase	TECR			++			+			++	-0.36741	-0.0433581	1.72703	-0.597398	-0.973678	0.814787	-1.1265	-0.577615	1.14414
ATP-dependent 6-phosphofructokinase, muscle type	PFKM			++			++			++	0.308483	0.256879	1.00538	-0.737245	-0.501066	1.03588	-1.73446	-0.787765	1.15391
Transmembrane protein 160	TMEM160				++	+			++		-1.29843	-0.826072	-1.15441	1.02186	0.955177	-0.570521	0.470212	1.33859	0.0635903
60S ribosomal protein L14	RPL14		++				+	+			-0.178068	1.59086	-0.857874	0.437768	-0.872996	0.909551	0.9638	-0.944434	-1.04861
Ribonuclease H2 subunit A	RNASEH2A	+		+			++			+	0.865022	-0.504496	0.83025	-0.569627	-0.934648	1.43693	-0.991628	-1.07908	0.947281
Nuclear pore complex protein Nup214	NUP214			+	+		++			+	-1.14913	-0.15419	0.583942	0.922219	0.0707376	1.3922	-1.58182	-0.754489	0.67053
Transmembrane protein 33	TMEM33			++			+			++	-0.236059	0.158857	1.5536	-0.36982	-0.664922	0.867552	-1.43121	-0.968086	1.09009
DnaJ homolog subfamily B member 6	DNAJB6			++			+			++	0.225855	-0.141094	1.27199	-0.564934	-1.08313	0.989041	-1.03287	-0.998102	1.33325
45 kDa calcium-binding protein	SDF4	++					+			+	1.69674	-1.15838	0.295308	0.370548	-1.28859	0.846952	-0.523979	-0.780312	0.541721
Stromal cell-derived factor 2	SDF2	+++									2.57437	-0.720716	-0.291806	-0.106431	-0.256259	0.133288	-0.677046	-0.335522	-0.319876
Palmitoyl-protein thioesterase 1	PPT1	+		+			++			++	0.748236	-0.904832	0.768282	-0.323107	-1.27722	1.23909	-0.454137	-1.02357	1.22727
Transmembrane protein 126A	TMEM126A			++			+			++	-0.611812	-0.499041	1.72164	-0.679792	-0.987486	0.811577	-0.589764	-0.482807	1.31748
GTPase Era, mitochondrial	ERAL1	+		++			++			++	0.953552	-0.830814	1.04216	-0.786245	-0.857118	1.07203	-1.04094	-0.678722	1.1261
Dehydrogenase/reductase SDR family member 7B	DHRS7B			++						++	-0.672531	-0.393823	1.82807	-0.722979	-0.496684	0.136339	-0.72857	-0.519069	1.56925
Fatty acyl-CoA reductase 1	FAR1			++						++	-0.593133	-0.728035	1.76273	-0.380685	-0.803374	0.461095	-0.606364	-0.615895	1.50366
Zinc finger with UFM1-specific peptidase domain protein	ZUFSP	+					+		+	++	0.54132	-1.08307	-0.0827647	-0.970248	-0.80269	0.883176	-0.88806	0.707639	1.6947
Apolipoprotein L2	APOL2			++			++			++	0.481755	-0.572286	1.04496	-0.74544	-1.12996	1.32014	-0.88874	-0.720489	1.21006
Ras-related protein Rab-8A	RAB8A			++			++			++	-0.691967	-0.269389	1.365	-0.585703	-0.657136	1.20285	-1.01341	-0.706042	1.35579
Glutaminyl-peptide cyclotransferase-like protein	QPCTL			++			+			++	-0.549273	-0.644059	1.7833	-0.703347	-0.918072	0.792884	-0.510568	-0.518692	1.26782
Long-chain fatty acid transport protein 4	SLC27A4			+++						++	-0.137425	-0.435891	2.01469	-0.3894	-0.836576	-0.171358	-0.722461	-0.685291	1.36371
Alpha-1,6-mannosyl-glycoprotein 2-beta-N-acetylglucosaminyltransferase	MGAT2			++			+			++	-0.459095	-0.703822	1.302	-0.718092	-0.793968	0.902132	-0.586676	-0.64073	1.69825
Protein RER1	RER1			++			+			++	-0.940108	-0.018423	1.69468	-0.690342	-0.605902	0.64063	-0.878519	-0.575595	1.37358
Ufm1-specific protease 2	UFSP2						++			++	-0.146513	-0.366039	0.384494	-0.57931	-0.805029	1.87738	-0.92167	-0.766661	1.32335
Serine/threonine-protein kinase TBK1	TBK1	++					++			+	1.29677	-0.6004	-0.144344	-0.347909	-0.819611	1.16603	-1.70188	0.239918	0.911429
Unconventional myosin-VI	MYO6				++	++			++		-0.825606	-0.809204	-1.04394	1.04754	1.66123	-0.489618	-0.583951	1.0656	-0.0220613
TRPM8 channel-associated factor 1	TCAF1				+	+	+		+	+	-1.24685	-1.1802	-1.45249	0.660971	0.873428	0.806487	0.0704412	0.664026	0.804182
Dimethyladenosine transferase 2, mitochondrial	TFB2M			++			++			++	-0.0193515	-0.773511	1.2513	-0.468325	-1.2189	1.14624	-0.952591	-0.311126	1.34626
Probable ATP-dependent RNA helicase DDX41	DDX41						++	+	+		-1.55261	-0.586409	-1.3532	0.346343	0.214783	1.43504	0.690346	0.84319	-0.0374725
Ras-related protein Ral-A	RALA			++			++			++	-0.756294	-0.512702	1.01437	-0.784098	-0.523392	1.08653	-0.502027	-0.802901	1.78052
Peroxisomal membrane protein PEX16	PEX16			++			+			++	-0.918055	-0.409863	1.63444	-0.642584	-0.438271	0.52648	-0.611067	-0.731002	1.58992
Vesicle-trafficking protein SEC22b	SEC22B			++			++			++	-0.62952	0.284172	1.3596	-0.577102	-1.10848	1.08903	-0.874292	-0.786111	1.24271
Cancer-related nucleoside-triphosphatase	NTPCR	+		++			++			++	0.635102	-0.378438	1.22222	-0.754717	-1.15843	1.02705	-1.28401	-0.397903	1.08913
Ras-related protein Rab-9A	RAB9A			++			++			++	-0.67948	-0.379823	1.09438	-0.932883	-0.650901	1.57307	-0.563743	-0.721424	1.26081
DNA polymerase epsilon subunit 4	POLE4			++			++				-0.254993	-1.01291	1.71317	-0.648915	-0.312377	1.49981	-0.692749	-0.730201	0.439166
Ras-related protein Rab-35	RAB35			++			++			++	-0.611495	-0.501024	1.27821	-0.612982	-0.837859	1.08729	-0.506844	-0.867498	1.57221
Phosphorylase b kinase regulatory subunit beta	PHKB			++						++	-0.542211	-0.418479	1.63823	-0.579634	-0.421039	-0.190821	-0.418267	-0.891822	1.82404
E3 ubiquitin-protein ligase HECTD1	HECTD1				+	+		++	+		-1.39481	-1.2176	-1.32074	0.750972	0.646707	0.451646	1.03091	0.63674	0.416182
DnaJ homolog subfamily B member 11	DNAJB11	+++									2.03379	-1.39512	0.217462	0.228408	-1.00357	0.457969	0.0667665	-0.757305	0.151603
Protein NipSnap homolog 2	GBAS				++			++	++		-1.30286	-1.03877	-0.848874	1.21006	0.401672	-0.65856	1.18328	1.02098	0.0330789
WD and tetratricopeptide repeats protein 1	WDTC1	++	+	+		+	+				1.14531	0.747759	0.751562	0.0998887	0.52946	0.547301	-1.33777	-1.46634	-1.01717
ATP synthase subunit gamma, mitochondrial	ATP5C1			++			++			++	0.468566	-0.841733	1.11568	-0.14974	-1.50408	1.05687	-0.8206	-0.535504	1.21053
Target of Myb protein 1	TOM1					++		+	++		-0.952033	-1.12127	-1.21226	0.373438	1.07393	-0.457117	0.7544	1.51502	0.0258909
Malectin	MLEC			+			++			++	0.473748	-0.747781	0.98121	-0.802803	-0.928112	1.40564	-0.916293	-0.678723	1.21311
E3 ubiquitin-protein ligase RNF123	RNF123					+			++	+	-0.901479	-1.18345	-1.19575	0.0740316	0.873785	-0.0809568	0.0132563	1.79185	0.608717
Phenylalanine--tRNA ligase alpha subunit	FARSA			++			+			++	0.259247	-0.432107	1.38147	-1.08789	-0.905855	0.897441	-0.996509	-0.46459	1.34879
Cyclin-dependent kinase 1	CDK1;CDC2			++			+			++	0.463968	-0.196418	1.004	-0.394632	-1.54212	0.93647	-0.110037	-1.36556	1.20433
Tricarboxylate transport protein, mitochondrial	SLC25A1			++			+			++	0.174923	-0.378447	1.55265	-0.76317	-1.15219	0.698974	-0.99273	-0.481489	1.34148
Protein NipSnap homolog 1	NIPSNAP1				++			++	++		-1.23721	-1.21474	-0.740683	1.02767	0.305227	-0.393878	1.21315	1.27112	-0.230658
Ubiquitin-associated domain-containing protein 2	UBAC2						+	+	+	++	-1.65078	-1.44255	0.0604404	0.413396	-0.474132	0.623687	0.595057	0.554057	1.32082
NADH dehydrogenase [ubiquinone] iron-sulfur protein 2, mitochondrial	NDUFS2	++		+						++	1.30754	0.0808584	0.539453	-0.685958	-0.490484	0.4815	-0.685392	-1.76208	1.21456
Sharpin	SHARPIN					++			++		-0.90601	-1.29369	-1.48416	0.262362	1.04332	0.443899	0.430706	1.31416	0.189402
FAS-associated factor 2	FAF2			++			++			++	-1.21278	-0.344576	1.0326	-0.856756	-0.80167	1.31991	-0.623021	0.137904	1.34838
LanC-like protein 1	LANCL1	++		++			++				1.5559	-0.687365	1.15973	-0.0408144	-0.44416	1.00973	-0.905802	-1.25143	-0.395793
Uncharacterized protein NCBP2-AS2	NCBP2-AS2			++						++	-0.732794	-0.536292	1.7738	-0.56321	-0.635319	0.277751	-0.611773	-0.5651	1.59294
TBC1 domain family member 15	TBC1D15				+	+	+	+	+	+	-1.47934	-1.08629	-1.40352	0.764443	0.696947	0.549818	0.687196	0.566153	0.704602
Trifunctional enzyme subunit alpha, mitochondrial;Long-chain enoyl-CoA hydratase;Long chain 3-hydroxyacyl-CoA dehydrogenase	HADHA				+	++		++	+		-0.842556	-0.803873	-1.17075	0.894153	1.15025	-0.864038	1.12945	0.960687	-0.453323
RuvB-like 1	RUVBL1			+						++	0.0633086	0.00739591	0.568172	-1.60396	-1.24336	0.32957	-0.195571	0.244773	1.82967
Coronin;Mitochondrial import inner membrane translocase subunit TIM16	PAM16;CORO7-PAM16			++			+			++	-0.734778	-0.778658	1.3232	-0.374468	-0.666982	0.732101	-1.03948	-0.157016	1.69609
Ubiquitin carboxyl-terminal hydrolase 15	USP15				+		++			++	-1.19386	-0.870468	-0.461501	0.672658	0.443691	1.39284	-0.858825	-0.535373	1.41084
40S ribosomal protein S5;40S ribosomal protein S5, N-terminally processed	RPS5			++			+			++	-0.625094	-0.129723	1.18233	0.0185727	-1.7922	0.89089	-0.627714	-0.235735	1.31867
Insulin receptor substrate 4	IRS4			++			+			++	-0.0689247	-0.25887	1.26598	-0.41349	-0.525084	0.956122	-1.64765	-0.656673	1.34859
Ubiquitin thioesterase ZRANB1	ZRANB1				++	+			+		-1.65607	-0.631005	-1.40149	1.15858	0.762656	0.292512	0.494545	0.773425	0.206857
Very-long-chain 3-oxoacyl-CoA reductase	HSD17B12			++			+			++	-0.134217	-0.818954	1.47001	-0.40734	-1.16788	0.873279	-1.01328	-0.146831	1.34521
Ubiquitin thioesterase OTUB1	OTUB1				+			++	++		-0.964226	-0.455448	-0.83327	0.909293	-0.735611	-0.845965	1.7124	1.06309	0.149736
RanBP-type and C3HC4-type zinc finger-containing protein 1	RBCK1					++			++		-1.24642	-1.04358	-1.29263	0.167604	1.24833	0.315362	0.197195	1.37189	0.282241
Mitochondrial 2-oxoglutarate/malate carrier protein	SLC25A11			++						++	0.464072	-0.162809	1.52083	-0.23664	-1.80368	0.489119	-0.488716	-0.795013	1.01283
E3 ubiquitin-protein ligase UBR5	UBR5						++		+	++	-1.29406	-1.31476	-0.487401	0.287763	0.353131	1.15106	-0.694343	0.582485	1.41612
Armadillo repeat-containing X-linked protein 3	ARMCX3			++			+			++	-0.351049	-0.684183	1.32392	-0.189999	-0.952167	0.918387	-0.896747	-0.750187	1.58203
Trifunctional enzyme subunit beta, mitochondrial;3-ketoacyl-CoA thiolase	HADHB				+	+		++	++		-1.33543	-0.67134	-1.39002	0.785283	0.877332	-0.400098	1.02069	1.11602	-0.00244686
RuvB-like 2	RUVBL2									+++	0.0456911	-0.1091	0.0287027	-0.513615	-1.66918	0.269103	-0.338246	0.110362	2.17628
Heme oxygenase 1	HMOX1			++			++			++	0.473768	-0.519168	1.05507	-0.875383	-1.30274	1.24833	-0.915208	-0.351938	1.18728
Calumenin	CALU	+++					+				2.05694	-0.814982	-0.220694	0.0103207	-0.975672	0.887791	-0.562417	-0.87141	0.490126
Reticulocalbin-1	RCN1	++					+			+	1.97419	-0.909226	-0.287081	0.232015	-0.870016	0.85783	-0.729977	-0.868079	0.600343
Stromal cell-derived factor 2-like protein 1	SDF2L1	+++					+				2.27551	-0.654388	0.0579218	0.103893	-0.841465	0.689806	-0.51937	-0.163612	-0.948297
NADH dehydrogenase [ubiquinone] 1 alpha subcomplex subunit 9, mitochondrial	NDUFA9			++			+			++	-0.549623	-0.299374	1.30278	-0.62201	-1.07614	0.996472	-0.823016	-0.494681	1.56559
Tax1-binding protein 1	TAX1BP1				+	+		+	+		-1.20623	-1.20998	-1.29051	0.804363	0.98618	0.0845858	0.978791	0.909702	-0.0568993
Dolichol-phosphate mannosyltransferase subunit 1	DPM1			++			+			++	0.410794	-0.729672	1.30267	-0.753462	-1.07977	0.974805	-1.00035	-0.438438	1.31342
E3 ubiquitin-protein ligase RNF31	RNF31					++	+		++		-1.11526	-0.96196	-1.58634	0.312455	1.18707	0.50643	0.224987	1.2033	0.22931
Ubiquitin-associated domain-containing protein 1	UBAC1					+			++	+	-1.00346	-1.05452	-0.985379	-0.185885	0.861343	-0.252222	0.154448	1.94219	0.52348
Mitochondrial import inner membrane translocase subunit TIM14	DNAJC19			++						++	-0.863458	-0.620346	1.56145	-0.549164	-0.464883	0.00607757	-0.451262	-0.483031	1.86462
Phosphatidylglycerophosphatase and protein-tyrosine phosphatase 1	PTPMT1			++			+			++	-0.714431	-0.710734	1.36818	-0.665753	-0.616108	0.729858	-0.598315	-0.551823	1.75913
Ubiquitin carboxyl-terminal hydrolase isozyme L1;Ubiquitin carboxyl-terminal hydrolase	UCHL1				++			++	+		-1.29754	-0.194561	-1.5692	1.29713	-0.11858	0.224391	1.23904	0.681914	-0.262592
ATPase WRNIP1	WRNIP1				+	+	+	+	+		-1.53649	-0.715958	-1.55991	0.867999	0.79952	0.537323	0.703842	0.684427	0.21925
Proliferating cell nuclear antigen	PCNA	++						+		+	1.32654	0.0957009	0.138956	-0.237262	-2.26256	0.0664268	0.548162	-0.420624	0.744666
Acylglycerol kinase, mitochondrial	AGK			++			+			++	-0.0781761	-0.535252	1.28269	-0.510972	-1.03992	0.791191	-1.09243	-0.457526	1.64039
Ubiquitin carboxyl-terminal hydrolase 5	USP5				++	+		+	+		-1.40845	-0.513727	-1.40093	1.06411	0.995454	0.165247	0.893259	0.742923	-0.537889
TOM1-like protein 2	TOM1L2				+	++		+	++		-1.15768	-1.23927	-1.26232	0.703292	1.12404	0.229475	0.78577	1.02838	-0.211685
Cytochrome c oxidase subunit 6C	COX6C			++						++	-0.810313	-0.285158	1.55367	-0.80976	-0.608093	0.339448	-0.904339	-0.167116	1.69166
Tubulin beta-4A chain	TUBB4A	++		+			+			++	1.34752	-0.429801	0.779772	-1.05255	-1.40743	0.63144	-0.376938	-0.649572	1.15756
TIP41-like protein	TIPRL		+	+++							-0.345543	0.7232	2.3605	-0.216968	0.0612122	-0.427704	-0.664845	-0.746229	-0.743626
Phospholipase A-2-activating protein	PLAA	++	+	++							1.46294	0.875863	1.13696	-0.252493	-0.594991	0.150216	-1.57741	-0.874543	-0.326533
Ubiquitin carboxyl-terminal hydrolase 13	USP13				++	+		+	+		-1.28104	-1.0535	-1.32017	1.00744	0.895882	0.14487	0.947405	0.855358	-0.196249
Sequestosome-1	SQSTM1				+	+		+	+		-0.841326	-1.02272	-1.79753	0.756664	0.914614	0.115508	0.867801	0.911297	0.0956931

		GO-Annotations																	
Protein names	Gene names	GOBP name	GOMF name	GOCC name	Peptides	Razor + unique peptides	Unique peptides	Sequence coverage [%]	Unique + razor sequence coverage [%]	Unique sequence coverage [%]	Mol. weight [kDa]	Q-value	Score	Intensity	MS/MS count	-Log ANOVA p value	ANOVA q-value	Protein IDs	Majority protein IDs
Sortilin	SORT1	biological regulation;carbohydrate transport;cell differe	binding;enzyme binding;growth factor binding;n	cell part;cell surface;clathrin-coated vesic	9	9	9	14.9	14.9	14.9	92.067	0	67.541	364420000	75	13.5161	0.00369231	Q99523;Q99523-2;A0A0J9YVX1;A0A0J9YX61	Q99523;Q99523-2
Neuralized-like protein 4	NEURL4			cell part;centriole;cytoplasmic part;cytos	17	17	17	15.4	15.4	15.4	164.31	0	39.982	282350000	40	9.30066	0.00924675	I3L2W2;Q96JN8-2;Q96JN8;I3L100;I3L2Z9;K7EPS7;K7ESJ6;K7ESM1	I3L2W2;Q96JN8-2;Q96JN8;I3L100;I3L2Z9
E3 ubiquitin-protein ligase HERC2	HERC2	biological regulation;cellular macromolecule metabolic p	acid-amino acid ligase activity;binding;catalytic a	cell part;centriole;cytoplasm;cytoplasmic	58	58	58	17.4	17.4	17.4	527.22	0	270.29	1581200000	265	13.7943	0.00230303	O95714;A0A0J9YVP0;A0A0J9YXQ8;Q9BVR0;A0A0G2JPS8;H3BUQ1	O95714;A0A0J9YVP0
ADP-dependent glucokinase	ADPGK	alcohol catabolic process;alcohol metabolic process;carb	ADP-specific glucokinase activity;binding;catalyt	cell part;cytoplasmic part;endoplasmic re	7	7	7	18.3	18.3	18.3	53.96	0	98.479	150930000	25	12.2565	0.0109398	Q9BRR6-2;Q9BRR6;H3BRS6;Q9BRR6-4;Q9BRR6-3;H3BTH4;H3BN66;Q9BRR6-6	Q9BRR6-2;Q9BRR6;H3BRS6
Mitochondrial import receptor subunit TOM5 homolog	TOMM5	cellular macromolecule metabolic process;cellular metabolic process;cellular process;cellular protein meta		cell part;cytoplasmic part;integral to men	4	4	4	18.4	18.4	18.4	10.976	0	9.2629	824560000	26	9.2051	0.00846377	F8W8Z9;Q8N4H5;Q8N4H5-3;Q8N4H5-2;H3BUX3;H0YFG9	F8W8Z9;Q8N4H5;Q8N4H5-3;Q8N4H5-2
von Willebrand factor A domain-containing protein 8	VWA8		adenyl nucleotide binding;adenyl ribonucleotide	cell part;cytoplasmic part;extracellular re	24	24	24	18.9	18.9	18.9	210.92	0	153.22	343440000	60	10.155	0.00938667	A3KMH1-3;A3KMH1;A3KMH1-2	A3KMH1-3;A3KMH1;A3KMH1-2
Delta(24)-sterol reductase	DHCR24;Nbla03646	alcohol metabolic process;amyloid precursor protein ca	antigen binding;binding;catalytic activity;coenzy	cell part;cytoplasmic part;cytoskeleton;cy	9	9	9	20.5	20.5	20.5	60.101	0	34.113	466090000	35	8.47207	0.01328	Q15392;Q3LIE7;Q15392-2;H7C4B7	Q15392;Q3LIE7;Q15392-2
Probable serine carboxypeptidase CPVL	CPVL		carboxypeptidase activity;catalytic activity;exope	extracellular membrane-bounded organe	9	9	9	21	21	21	54.163	0	44.262	524560000	80	8.91876	0.0127556	Q9H3G5;H7C0X5;C9JLV0;H7C218;C9J6L4;C9JI22	Q9H3G5;H7C0X5
TAF6-like RNA polymerase II p300/CBP-associated factor-associated factor 65 kDa subunit 6L	TAF6L	biological regulation;biosynthetic process;cellular biosy	binding;DNA binding;nucleic acid binding;protein	cell part;chromatin remodeling complex;c	11	11	11	21.9	21.9	21.9	67.814	0	77.602	406160000	74	11.5508	0.00513333	Q9Y6J9;E9PP94	Q9Y6J9
Protein PET100 homolog, mitochondrial	PET100			cell part;cytoplasmic part;integral to men	2	2	2	22.7	22.7	22.7	5.5534	0	7.4809	403380000	21	8.63441	0.0126122	A0A0A0MSK2;P0DJ07	A0A0A0MSK2;P0DJ07
Derlin-1	DERL1	biological regulation;catabolic process;cellular catabolic	binding;enzyme binding;MHC class I protein bin	cell part;cytoplasmic part;cytoplasmic ub	5	5	5	24.2	24.2	24.2	26.423	0	39.544	645400000	103	15.1274	0.002125	Q9BUN8-2;Q9BUN8;B4E1G1;E5RGY0	Q9BUN8-2;Q9BUN8;B4E1G1;E5RGY0
Phosphatidate cytidylyltransferase, mitochondrial	TAMM41	acylglycerol biosynthetic process;acylglycerol metabolic	catalytic activity;cytidylyltransferase activity;nuc	cell part;cytoplasmic part;extrinsic to me	8	8	8	25.5	25.5	25.5	38.078	0	149.04	359130000	55	11.1085	0.0104198	Q96BW9-3;Q96BW9;Q96BW9-2;A0A0G2JQ92;B4E101;H7C1A2;G3V0F3	Q96BW9-3;Q96BW9;Q96BW9-2;A0A0G2JQ92
Mammalian ependymin-related protein 1	EPDR1	biological adhesion;cell adhesion;cell-matrix adhesion;ce	binding;calcium ion binding;cation binding;ion bi	cell part;cytoplasmic part;extracellular m	6	6	6	29	29	29	25.437	0	62.897	682600000	88	8.1272	0.0138431	Q9UM22;Q9UM22-2;Q9UM22-3;D6RIH7	Q9UM22;Q9UM22-2;Q9UM22-3
Glutaryl-CoA dehydrogenase, mitochondrial	GCDH	acyl-CoA biosynthetic process;acyl-CoA metabolic proce	acyl-CoA dehydrogenase activity;binding;carbox	cell part;cytoplasmic part;intracellular me	8	8	8	29.2	29.2	29.2	48.127	0	285.75	793780000	107	14.6166	0.00228571	Q92947;Q92947-2;K7EKT3;K7EQ99;K7ESA6;K7ES74;K7EKH1	Q92947;Q92947-2
Heterogeneous nuclear ribonucleoprotein U-like protein 1	HNRNPUL1	biological regulation;biosynthetic process;cellular biosy	binding;enzyme binding;nucleic acid binding;pro	cell part;intracellular membrane-bounde	17	17	17	29.4	29.4	29.4	90.291	0	323.31	1045000000	118	10.3105	0.00926316	Q9BUJ2-2;Q9BUJ2;A0A0A0MRA5;B7Z4B8;Q9BUJ2-4;M0R3F1;Q9BUJ2-3;M0QYZ0;M0QYM5;M0R0K8;M0QYI8;M0R203;M0QZV6;Q9BUJ2-5;M0R247;A0A087X1I2	Q9BUJ2-2;Q9BUJ2;A0A0A0MRA5;B7Z4B8;Q9BUJ2-4;M0R3F1;Q9BUJ2-3;M0QYZ0
Protein kinase C iota type	PRKCI	actin cytoskeleton organization;actin filament organizati	adenyl nucleotide binding;adenyl ribonucleotide	apical plasma membrane;cell junction;cel	11	11	11	30	30	30	68.262	0	151.27	655520000	103	6.96304	0.0192212	P41743	P41743
DnaJ homolog subfamily A member 3, mitochondrial	DNAJA3	activation of caspase activity;activation-induced cell dea	adenyl nucleotide binding;adenyl ribonucleotide	cell junction;cell part;cytoplasm;cytoplas	13	13	2	30.4	30.4	2.7	52.488	0	193.61	1733700000	211	14.7758	0.003	Q96EY1;I3L1T6	Q96EY1
Cytochrome c oxidase subunit NDUFA4	NDUFA4	cellular metabolic process;cellular process;electron tran	binding;catalytic activity;NADH dehydrogenase (	cell part;cytoplasmic part;extracellular m	3	3	3	30.9	30.9	30.9	9.3697	0	9.7444	688400000	28	7.58426	0.0176449	O00483	O00483
E3 ubiquitin-protein ligase MIB2	MIB2	biological regulation;cell surface receptor linked signalin	acid-amino acid ligase activity;binding;catalytic a	cell part;cytoplasm;cytoplasmic part;earl	21	21	21	31	31	31	105.15	0	285.78	1069600000	138	13.9563	0.00235294	E9PD12;Q96AX9-2;Q96AX9;Q96AX9-11;Q96AX9-14;F2Z2L2;Q96AX9-4;Q96AX9-6;Q96AX9-12;Q96AX9-3;Q96AX9-13;D6RAZ0;Q96AX9-8;Q96AX9-7;Q96AX9-5;Q96AX9-10;H0YAM8;Q96AX9-9;D6RED3;D6RFJ2;D6RE96;J3QR95;D6R9Q6	E9PD12;Q96AX9-2;Q96AX9;Q96AX9-11;Q96AX9-14;F2Z2L2;Q96AX9-4;Q96AX9-6;Q96AX9-12;Q96AX9-3;Q96AX9-13;D6RAZ0;Q96AX9-8;Q96AX9-7;Q96AX9-5;Q96AX9-10;H0YAM8;Q96AX9-9
CUE domain-containing protein 1	CUEDC1				7	7	7	31.3	31.3	31.3	42.258	0	25.078	199530000	34	10.2151	0.0132929	Q9NWM3;J3QLQ8;Q9NWM3-2;J3KT42;V9GY27;B1AQP5;O75618;O75618-2	Q9NWM3;J3QLQ8;Q9NWM3-2
Lethal(2) giant larvae protein homolog 1	LLGL1	actin cytoskeleton organization;actin filament-based pro	binding;enzyme activator activity;enzyme bindin	axon;cell cortex part;cell part;cell projecti	22	22	22	31.7	31.7	31.7	115.42	0	132.29	1776500000	218	10.6804	0.0042807	Q15334;A0A087WW77	Q15334;A0A087WW77
Very-long-chain enoyl-CoA reductase	TECR	acyl-CoA biosynthetic process;acyl-CoA metabolic proce	catalytic activity;oxidoreductase activity;oxidore	cell part;cytoplasmic part;endoplasmic re	13	13	13	32.5	32.5	32.5	36.034	0	247.9	6211200000	448	10.8398	0.012701	Q9NZ01;M0R3C3;Q9NZ01-2;M0QXM3;M0R329;B4DR74	Q9NZ01
ATP-dependent 6-phosphofructokinase, muscle type	PFKM	alcohol catabolic process;alcohol metabolic process;ana	6-phosphofructokinase activity;adenyl nucleotid	6-phosphofructokinase complex;apical pl	19	19	16	32.6	32.6	29.1	85.182	0	134.7	796770000	94	8.44089	0.0143301	P08237;P08237-3;P08237-2;F8VX13;F8VNX2;F8VZQ1;F8VP00;F8W1J8;F8VSF7;F8VW30;F8VSL1;F8VUB8;F8VYK8;F8VTQ3;F8VZI0;F8VVE3	P08237;P08237-3;P08237-2
Transmembrane protein 160	TMEM160			cell part;cytoplasmic part;integral to men	6	6	6	33	33	33	19.657	0	323.31	1902700000	174	11.7306	0.00309091	Q9NX00	Q9NX00
60S ribosomal protein L14	RPL14	biosynthetic process;catabolic process;cellular biosynth	binding;nucleic acid binding;RNA binding;structu	cell part;cytoplasm;cytoplasmic part;cyto	4	4	4	33.1	33.1	33.1	14.558	0	15.93	509680000	63	9.12084	0.0127191	E7EPB3;P50914	E7EPB3;P50914
Ribonuclease H2 subunit A	RNASEH2A	biosynthetic process;catabolic process;cellular biosynth	binding;catalytic activity;cation binding;endonuc	cell part;intracellular membrane-bounde	9	9	9	33.4	33.4	33.4	33.395	0	127.43	483480000	81	19.0716	0.00145455	O75792;A0A1W2PP97	O75792
Nuclear pore complex protein Nup214	NUP214	biological regulation;biosynthetic process;carbohydrate	nucleocytoplasmic transporter activity;transport	adherens junction;anchoring junction;cel	45	45	45	34.5	34.5	34.5	212.57	0	323.31	2344600000	288	9.91536	0.0090411	P35658-2;P35658-4;P35658;P35658-3;P35658-5;A0A0A0MSW3;H0Y837;B7ZAV2;H0YF36;E9PKD2;H0YDS4	P35658-2;P35658-4;P35658;P35658-3;P35658-5;A0A0A0MSW3
Transmembrane protein 33	TMEM33	cellular component assembly;cellular component assem	structural constituent of nuclear pore;structural	cell part;cytoplasmic membrane-bounde	8	8	8	35.2	35.2	35.2	27.978	0	130.07	3468300000	188	13.6263	0.00407143	P57088;D6RAA6;H0Y8N0;J3KN43;D6RBY2	P57088;D6RAA6
DnaJ homolog subfamily B member 6	DNAJB6	actin cytoskeleton organization;actin filament-based pro	ATPase activator activity;ATPase regulator activi	cell part;contractile fiber part;cytoplasm;	7	7	6	35.5	35.5	31.1	25.377	0	45.53	569590000	67	11.4313	0.00609231	C9J2C4;A0A0J9YX62;O75190-2;O75190-3;O75190;C9JDX6;F8WCZ4;C9J2P2;E9PH18;C9JDR7;C9JB42;C9JN01;O75190-4;Q8WWF6;Q7Z6W7	C9J2C4;A0A0J9YX62;O75190-2;O75190-3;O75190;C9JDX6;F8WCZ4;C9J2P2;E9PH18;C9JDR7;C9JB42
45 kDa calcium-binding protein	SDF4	anatomical structure development;calcium ion-depende	binding;calcium ion binding;cation binding;identi	bleb;cell part;cell projection;cytoplasm;cy	10	10	10	35.9	35.9	35.9	41.806	0	97.553	733330000	116	9.69094	0.0116235	Q9BRK5;Q9BRK5-6;G3V1E2;Q9BRK5-4;Q9BRK5-3;Q9BRK5-2;H0Y3T6;Q9BRK5-5	Q9BRK5;Q9BRK5-6;G3V1E2;Q9BRK5-4;Q9BRK5-3;Q9BRK5-2
Stromal cell-derived factor 2	SDF2	carbohydrate metabolic process;cellular carbohydrate n	catalytic activity;dolichyl-phosphate-mannose-p	cell part;extracellular region part;extracel	6	6	6	37	37	37	23.026	0	158.68	283400000	29	14.3963	0.00368627	Q99470;K7ELI9	Q99470
Palmitoyl-protein thioesterase 1	PPT1	adult behavior;adult locomotory behavior;anatomical st	acyl-CoA thioesterase activity;catalytic activity;C	axon;cell body;cell part;cell projection;cla	9	9	9	37	37	37	34.136	0	198.02	825980000	125	14.542	0.00246154	A0A2C9F2P4;A0A286YFF7;P50897;Q5T0S4;A0A286YFE3;A0A286YFL8;P50897-2;E9PK48;E9PIA8;A0A286YFL6;E9PSE5	A0A2C9F2P4;A0A286YFF7;P50897;Q5T0S4;A0A286YFE3;A0A286YFL8;P50897-2
Transmembrane protein 126A	TMEM126A	anatomical structure development;cranial nerve development;developmental process;nerve developmen		cell part;cytoplasmic part;integral to men	8	8	8	37.9	37.9	37.9	21.527	0	21.27	405560000	37	8.35162	0.0174679	Q9H061;E9PI90;Q9H061-2;E9PIH8	Q9H061;E9PI90;Q9H061-2
GTPase Era, mitochondrial	ERAL1	cellular component assembly;cellular component assem	binding;GTP binding;guanyl nucleotide binding;g	actin cytoskeleton;cell part;cytoplasm;cy	12	12	12	38.2	38.2	38.2	48.349	0	209.35	652980000	123	15.2601	0.00222222	O75616;J3QSB2;J3QRV9;O75616-2;J3QT61;K7EQJ8	O75616;J3QSB2;J3QRV9;O75616-2
Dehydrogenase/reductase SDR family member 7B	DHRS7B		catalytic activity;oxidoreductase activity	cell part;cytoplasmic part;endoplasmic re	9	9	9	38.7	38.7	38.7	33.524	0	66.293	310000000	46	10.5358	0.0131485	A0A0C4DGQ8;Q6IAN0;J3KRS1;J3QLK1;J3QLJ8;J3QKT1	A0A0C4DGQ8;Q6IAN0;J3KRS1
Fatty acyl-CoA reductase 1	FAR1	acyl-CoA metabolic process;biosynthetic process;carbox	catalytic activity;fatty-acyl-CoA reductase (alcoh	cell part;cytoplasmic part;integral to men	17	17	17	38.8	38.8	38.8	59.356	0	138.23	1023100000	106	11.0337	0.00716418	Q8WVX9;E9PNW8;E9PPB8;A0A087X0B3;P33260-2;P33260	Q8WVX9;E9PNW8
Zinc finger with UFM1-specific peptidase domain protein	ZUFSP		binding;cation binding;ion binding;metal ion binding		19	19	19	38.9	38.9	38.9	65.958	0	173.71	1767300000	185	7.80451	0.0124167	Q96AP4;X6R6X3;Q96AP4-2	Q96AP4
Apolipoprotein L2	APOL2	acute inflammatory response;acute-phase response;alc	binding;high-density lipoprotein particle binding	cell part;cytoplasmic part;endoplasmic re	12	12	1	39.5	39.5	4.2	37.092	0	100.98	919280000	119	17.1468	0.00215385	Q9BQE5;E9PM95;B0QYK8	Q9BQE5;E9PM95
Ras-related protein Rab-8A	RAB8A	anatomical structure formation involved in morphogene	binding;catalytic activity;cytoskeletal protein bin	cell body;cell part;cell projection;centros	8	6	5	39.6	30.9	23.7	23.668	0	128.48	309310000	31	10.6272	0.0129655	P61006;P61006-2	P61006;P61006-2
Glutaminyl-peptide cyclotransferase-like protein	QPCTL	cellular macromolecule metabolic process;cellular metab	binding;catalytic activity;cation binding;glutamin	cell part;cytoplasmic part;Golgi apparatu	15	15	15	39.8	39.8	39.8	42.924	0	137.59	1069700000	71	9.78782	0.00916667	Q9NXS2;Q9NXS2-3;K7EQG1	Q9NXS2;Q9NXS2-3
Long-chain fatty acid transport protein 4	SLC27A4	anatomical structure development;carboxylic acid catab	binding;catalytic activity;fatty acid ligase activity;	brush border membrane;cell part;cell pro	21	21	21	40	40	40	72.063	0	198.58	596700000	62	7.78166	0.0176296	Q6P1M0;Q6P1M0-2	Q6P1M0
Alpha-1,6-mannosyl-glycoprotein 2-beta-N-acetylglucosaminyltransferase	MGAT2	biosynthetic process;carbohydrate biosynthetic process	acetylglucosaminyltransferase activity;alpha-1,6	cell part;cytoplasmic part;Golgi apparatu	15	15	15	40.5	40.5	40.5	51.55	0	323.31	1326200000	89	17.6475	0.002	Q10469	Q10469
Protein RER1	RER1	biological regulation;cellular component organization;cellular component organization or biogenesis;cellu		cell part;cell surface;cytoplasmic part;enc	8	8	8	41.3	41.3	41.3	22.958	0	90.429	798200000	108	15.167	0.00219355	O15258;Q5T092;A0A0A0MR06;Q5T091;Q5T093	O15258;Q5T092;A0A0A0MR06;Q5T091;Q5T093
Ufm1-specific protease 2	UFSP2	biological regulation;macromolecule metabolic process;	catalytic activity;cysteine-type peptidase activity	cell part;cytoplasm;cytoplasmic part;end	14	14	14	41.4	41.4	41.4	53.261	0	192.42	499970000	66	8.67004	0.0124632	Q9NUQ7;D6RGX2;H0YA18;D6RB53;D6R9J7;D6RA67;H0Y9B0	Q9NUQ7;D6RGX2;H0YA18
Serine/threonine-protein kinase TBK1	TBK1	activation of immune response;activation of innate imm	adenyl nucleotide binding;adenyl ribonucleotide	cell part;cytoplasm;cytoplasmic part;cyto	23	23	23	41.7	41.7	41.7	83.641	0	251.41	1527700000	164	9.60415	0.00902703	Q9UHD2;F5H206;F5H1A3;F5GZI4;A0A075B7B4;O14164-2;O14164	Q9UHD2
Unconventional myosin-VI	MYO6	actin filament-based movement;actin filament-based pro	actin binding;actin filament binding;adenyl nucle	apical part of cell;axon;cell body;cell corte	44	44	44	41.8	41.8	41.8	145.01	0	323.31	2192300000	307	6.71245	0.0173091	A0A0A0MRM8;Q9UM54-6;A0A0D9SGC1;E7EW20;Q9UM54-5;Q9UM54-2;Q9UM54-4;Q9UM54-1;Q9UM54;Q5JVM0	A0A0A0MRM8;Q9UM54-6;A0A0D9SGC1;E7EW20;Q9UM54-5;Q9UM54-2;Q9UM54-4;Q9UM54-1;Q9UM54
TRPM8 channel-associated factor 1	TCAF1	biological regulation;establishment of localization;negati	binding;ion channel binding;protein binding	cell part;membrane;plasma membrane	30	30	30	42.1	42.1	42.1	102.12	0	323.31	3522100000	328	12.0275	0.0033913	Q9Y4C2;Q9Y4C2-2;C9K0E7;C9J9F4;C9JD13;A0A1B0GVM2;A6NFQ2-3;A6NFQ2	Q9Y4C2;Q9Y4C2-2
Dimethyladenosine transferase 2, mitochondrial	TFB2M	biological regulation;biosynthetic process;cellular biosy	catalytic activity;methyltransferase activity;N-m	cell part;cytoplasmic part;intracellular no	17	17	17	42.7	42.7	42.7	45.348	0	67.21	884240000	114	17.0329	0.00222222	Q9H5Q4	Q9H5Q4
Probable ATP-dependent RNA helicase DDX41	DDX41	apoptosis;biological regulation;cell death;cellular macro	adenyl nucleotide binding;adenyl ribonucleotide	catalytic step 2 spliceosome;cell part;cyto	24	24	24	43.4	43.4	43.4	71.648	0	130.43	1003600000	150	9.18125	0.00901408	J3KNN5;Q9UJV9;H0Y8L8;D6RGI7;H0YA06;D6RD33	J3KNN5;Q9UJV9
Ras-related protein Ral-A	RALA	actin cytoskeleton organization;actin cytoskeleton reorg	ATPase binding;binding;catalytic activity;cytoske	adherens junction;anchoring junction;cel	9	9	9	43.7	43.7	43.7	23.567	0	220.6	2529900000	75	15.591	0.00190476	P11233;H7C3P7;C9JPE8;P11234;P11234-3;P11234-2;C9JYR1;C9J6B1;C9JQB3;F8WEQ6	P11233;H7C3P7
Peroxisomal membrane protein PEX16	PEX16	cellular component biogenesis;cellular component bioge	binding;protein binding;protein C-terminus bind	cell part;cytoplasmic part;endoplasmic re	11	11	11	44	44	44	38.628	0	57.205	751740000	62	14.183	0.00316279	Q9Y5Y5;Q9Y5Y5-2;E9PMM3;E9PP98;E9PLS4;E9PQW0;E9PMS3	Q9Y5Y5;Q9Y5Y5-2;E9PMM3;E9PP98
Vesicle-trafficking protein SEC22b	SEC22B	biological regulation;cellular process;ER to Golgi vesicle-mediated transport;establishment of localization;		cell part;cytoplasmic membrane-bounde	8	8	8	44.2	44.2	44.2	24.593	0	74.396	303080000	66	15.205	0.00359184	O75396;A0A087X1A9	O75396
Cancer-related nucleoside-triphosphatase	NTPCR		adenyl nucleotide binding;adenyl ribonucleotide	cell part;extracellular membrane-bounde	7	7	7	44.3	44.3	44.3	25.072	0	143.48	782270000	144	11.5501	0.00571429	Q5TDF0;Q9BSD7	Q5TDF0;Q9BSD7
Ras-related protein Rab-9A	RAB9A	biological regulation;cellular process;cellular response to	binding;catalytic activity;GDP binding;GTP bindir	cell part;cytoplasmic membrane-bounde	6	6	5	44.8	44.8	39.3	22.837	0	89.783	147410000	17	10.6344	0.0126154	P51151;A0A1B0GUI0;REV__Q9NZZ3-2;REV__Q9NZZ3	P51151
DNA polymerase epsilon subunit 4	POLE4	biosynthetic process;cellular biosynthetic process;cellula	binding;catalytic activity;DNA binding;DNA polyr	Ada2/Gcn5/Ada3 transcription activator	3	3	3	45.3	45.3	45.3	12.209	0	46.942	287030000	33	12.7271	0.00468966	Q9NR33	Q9NR33
Ras-related protein Rab-35	RAB35	antigen processing and presentation;biological regulatio	binding;catalytic activity;GDP binding;GTP bindir	cell part;cell projection membrane;cell pr	8	8	8	45.3	45.3	45.3	23.025	0	105.8	386120000	42	16.0325	0.00227027	Q15286;F5H157;Q15286-2;F5H7F8	Q15286;F5H157;Q15286-2
Phosphorylase b kinase regulatory subunit beta	PHKB	alcohol metabolic process;carbohydrate catabolic proce	calmodulin-dependent protein kinase activity;ca	cell part;cytoplasmic part;cytosol;intrace	37	37	37	46	46	46	124.74	0	323.31	3490400000	142	6.66973	0.0198596	Q93100-3;Q93100-4;Q93100;Q93100-2;I3L213;H3BV13;H3BQ89;I3L3F2;I3L0Z1;I3L1N3;I3NHZ9	Q93100-3;Q93100-4;Q93100;Q93100-2
E3 ubiquitin-protein ligase HECTD1	HECTD1	anatomical structure formation involved in morphogene	acid-amino acid ligase activity;binding;catalytic a	cell part;cytoplasm;intracellular membra	90	90	0	46.1	46.1	0	289.38	0	323.31	1.3477E+10	1195	14.372	0.002	Q9ULT8	Q9ULT8
DnaJ homolog subfamily B member 11	DNAJB11	biological regulation;cellular macromolecule metabolic process;cellular metabolic process;cellular nitroger		cell part;cytoplasmic part;endoplasmic re	16	16	16	46.6	46.6	46.6	40.513	0	153.58	2139900000	196	9.39032	0.011381	Q9UBS4;H7C2Y5	Q9UBS4
Protein NipSnap homolog 2	GBAS	ATP biosynthetic process;ATP metabolic process;biological regulation;biosynthetic process;cellular biosyn		cell part;cytoplasmic part;integral to men	15	15	7	46.9	46.9	25.2	33.742	0	106.92	2374600000	143	10.8605	0.00407407	O75323;O75323-2;C9K068;C9J7B1;F8WBI5	O75323;O75323-2;C9K068
WD and tetratricopeptide repeats protein 1	WDTC1	alcohol metabolic process;biological regulation;carbohy	enzyme inhibitor activity;enzyme regulator activ	cell part;cytoplasmic part;cytosol;intrace	24	24	24	47.3	47.3	47.3	75.79	0	239.42	2324700000	321	11.9276	0.00557377	Q8N5D0-4;Q8N5D0;Q8N5D0-5;Q8N5D0-3;Q8N5D0-6;Q8N5D0-2;H0YEI6	Q8N5D0-4;Q8N5D0;Q8N5D0-5;Q8N5D0-3;Q8N5D0-6;Q8N5D0-2
ATP synthase subunit gamma, mitochondrial	ATP5C1	ATP biosynthetic process;ATP metabolic process;ATP sy	active transmembrane transporter activity;ATPa	cell part;cytoplasmic part;extracellular m	12	12	1	48	48	4.4	32.996	0	194.32	4323200000	216	8.38155	0.012093	P36542	P36542
Target of Myb protein 1	TOM1	cellular component organization;cellular component org	binding;clathrin binding;protein binding	cell part;cytoplasm;cytoplasmic part;cyto	15	15	15	48	48	48	53.818	0	202.58	861570000	192	8.27002	0.0124783	O60784;O60784-2;O60784-4;O60784-3;B0QY01;V9GYF4;B0QY02;Q6UW50;V9GZ68;H7BYN7;F8WB30;F8WAW7;F8WE29;F8WBB0	O60784;O60784-2;O60784-4;O60784-3;B0QY01
Malectin	MLEC	carbohydrate metabolic process;cellular carbohydrate n	binding;carbohydrate binding;enzyme binding;p	cell part;cytoplasmic part;endoplasmic re	12	12	12	48.6	48.6	48.6	32.233	0	323.31	1121600000	141	18.1905	0.00177778	Q14165;F5H1S8;H0YG07;F5GX14	Q14165;F5H1S8
E3 ubiquitin-protein ligase RNF123	RNF123	cellular macromolecule metabolic process;cellular metab	binding;catalytic activity;cation binding;ion bindi	cell part;cytoplasm;intracellular part	55	55	55	49.7	49.7	49.7	148.51	0	323.31	6512500000	431	13.1969	0.002	Q5XPI4;Q5XPI4-2;C9JS59;C9J266;C9JI97;F8WB91;C9JN91;H7C3U1	Q5XPI4;Q5XPI4-2;C9JS59;C9J266
Phenylalanine--tRNA ligase alpha subunit	FARSA	amine metabolic process;amino acid activation;carboxyl	adenyl nucleotide binding;adenyl ribonucleotide	cell part;cytoplasm;cytoplasmic part;cyto	19	19	19	50	50	50	57.563	0	323.31	1753100000	181	11.2505	0.00618182	Q9Y285;K7ER00;Q9Y285-2;K7EPH2;K7ER16;K7ERS0;K7EK06	Q9Y285;K7ER00;Q9Y285-2;K7EPH2
Cyclin-dependent kinase 1	CDK1;CDC2	activation of immune response;activation of innate imm	adenyl nucleotide binding;adenyl ribonucleotide	cell part;centrosome;cytoplasm;cytoplas	12	12	10	50.5	50.5	44.1	34.095	0	77.24	842560000	101	11.7688	0.006125	P06493;A0A024QZP7;A0A087WZZ9;E5RIU6;P06493-2;K7ELV5;G3V317;H0YAZ9;Q9BVE2;E7EUK8;A0A087X209;J3QSD7;Q00535-2;Q00535;Q00534;Q96Q40-2;P50750;Q96Q40-4;Q96Q40-3;O94921-3;Q96Q40-5;Q96Q40;O94921-2;O94921;P50750-2;Q9NYV4-3;Q14004-2;Q9NYV4-2;Q9NYV4;Q14004	P06493;A0A024QZP7;A0A087WZZ9;E5RIU6;P06493-2
Tricarboxylate transport protein, mitochondrial	SLC25A1	acyl-CoA biosynthetic process;acyl-CoA metabolic proce	carboxylic acid transmembrane transporter acti	cell part;cytoplasmic part;extracellular m	12	12	12	50.5	50.5	50.5	34.012	0	102.46	4421100000	233	8.78667	0.0128636	P53007;B4DP62	P53007;B4DP62
Protein NipSnap homolog 1	NIPSNAP1	multicellular organismal process;neurological system pr	binding;neurotransmitter binding	cell part;cytoplasmic part;intracellular me	14	13	13	50.7	48.6	48.6	33.31	0	205.36	2138000000	198	7.623	0.0125591	Q9BPW8;H7C2U6;C9JDV8;F8WCR5	Q9BPW8;H7C2U6
Ubiquitin-associated domain-containing protein 2	UBAC2	cellular localization;cellular macromolecule localization;cellular process;cellular protein localization;localiz		cell part;cytoplasmic part;endoplasmic re	15	15	15	50.9	50.9	50.9	38.963	0	237.79	2487400000	331	18.0482	0.0016	Q8NBM4;X6R5E5;Q8NBM4-3;Q8NBM4-2;Q8NBM4-4;Q5JUH4;Q8NBM4-5;A0A087WXT1	Q8NBM4;X6R5E5;Q8NBM4-3;Q8NBM4-2;Q8NBM4-4
NADH dehydrogenase [ubiquinone] iron-sulfur protein 2, mitochondrial	NDUFS2	cellular metabolic process;cellular process;electron tran	4 iron, 4 sulfur cluster binding;binding;catalytic a	cell part;cytoplasmic part;intracellular me	16	16	16	51	51	51	52.545	0	85.91	775840000	68	8.90473	0.0186667	O75306;O75306-2	O75306;O75306-2
Sharpin	SHARPIN	anatomical structure development;apoptotic nuclear ch	binding;cation binding;ion binding;metal ion bin	cell junction;cell part;cell projection;cyto	15	15	15	51.9	51.9	51.9	39.949	0	323.31	5630100000	283	14.7013	0.00211765	Q9H0F6;Q9H0F6-2;H0YCU2	Q9H0F6;Q9H0F6-2
FAS-associated factor 2	FAF2	biological regulation;cellular component organization;ce	binding;enzyme binding;enzyme inhibitor activit	Cdc48p-Npl4p-Ufd1p AAA ATPase compl	18	18	18	52.1	52.1	52.1	52.623	0	323.31	2216900000	133	15.2541	0.00243478	Q96CS3;D6RBG6	Q96CS3
LanC-like protein 1	LANCL1	biological regulation;cell surface receptor linked signalin	amine binding;amino acid binding;binding;carbo	cell part;cytoplasm;extracellular membra	15	15	15	52.6	52.6	52.6	45.283	0	74.502	1665500000	128	15.4935	0.0018	O43813;E9PHS0;F8WDS9;H7C2E3	O43813;E9PHS0
Uncharacterized protein NCBP2-AS2	NCBP2-AS2				5	5	5	53.5	53.5	53.5	10.89	0	35.945	1503000000	46	14.3712	0.00206897	Q69YL0	Q69YL0
TBC1 domain family member 15	TBC1D15	biological regulation;positive regulation of catalytic activ	enzyme activator activity;enzyme regulator activ	cell part;cytoplasm;cytoplasmic part;extr	33	33	33	54.2	54.2	54.2	77.394	0	323.31	5800100000	495	15.8626	0.002	Q8TC07-2;Q8TC07;Q8TC07-3;C9JA93;F8WCB5;F8VV61;F8WDJ1;F8WF35;F8WAX5	Q8TC07-2;Q8TC07;Q8TC07-3;C9JA93
Trifunctional enzyme subunit alpha, mitochondrial;Long-chain enoyl-CoA hydratase;Long chain 3-hydroxyacyl-CoA dehydrogenase	HADHA	biosynthetic process;carboxylic acid catabolic process;ca	3-hydroxyacyl-CoA dehydrogenase activity;acety	cell part;cytoplasmic part;fatty acid beta-	34	34	34	55.2	55.2	55.2	82.999	0	323.31	4147200000	575	9.34305	0.00847059	P40939;H0YFD6;P40939-2	P40939
RuvB-like 1	RUVBL1	ATP-dependent chromatin remodeling;biological regulat	5'-3' DNA helicase activity;adenyl nucleotide bin	cell part;chromatin remodeling complex;c	20	20	20	55.3	55.3	55.3	50.227	0	323.31	3446000000	208	16.9572	0.00186667	Q9Y265;Q9Y265-2;E7ETR0;H7C4G5;H7C4I3;J3QLR1	Q9Y265;Q9Y265-2;E7ETR0;H7C4G5
Coronin;Mitochondrial import inner membrane translocase subunit TIM16	PAM16;CORO7-PAM16	actin cytoskeleton organization;actin filament organizati	actin binding;binding;cytoskeletal protein bindin	cell part;cytoplasmic membrane-bounde	7	7	7	56.2	56.2	56.2	15.131	0	123.1	778030000	69	13.0858	0.00328889	I3L0X9;I3L3T0;P57737-3;A0A0A6YYL4;Q9Y3D7;I3L1U7;A0A0B4J298;Q9Y5T5-5;Q9Y5T5-4;Q9Y5T5-3;Q9Y5T5-2;Q9Y5T5;Q9ULL5-2;Q9ULL5;Q9ULL5-3	I3L0X9;I3L3T0;P57737-3;A0A0A6YYL4;Q9Y3D7;I3L1U7
Ubiquitin carboxyl-terminal hydrolase 15	USP15	biological regulation;BMP signaling pathway;catabolic p	binding;catalytic activity;cysteine-type endopept	cell part;cytoplasm;intracellular membra	45	45	44	56.2	56.2	55.2	112.42	0	323.31	2839900000	295	11.1372	0.004	Q9Y4E8;Q9Y4E8-2;Q9Y4E8-3;Q9Y4E8-4;H0YI31;F8W0H4;F8VZG8;F8VVY7;H0YI26;H0YHM4;H0YH96;Q13107-2;Q13107	Q9Y4E8;Q9Y4E8-2;Q9Y4E8-3
40S ribosomal protein S5;40S ribosomal protein S5, N-terminally processed	RPS5	biological regulation;biosynthetic process;catabolic proc	binding;mRNA binding;nucleic acid binding;RNA	adherens junction;anchoring junction;cel	13	13	13	56.5	56.5	56.5	22.391	0	222.58	5373400000	303	11.2422	0.0102278	M0R0F0;P46782;M0R0R2;M0QZN2	M0R0F0;P46782;M0R0R2;M0QZN2
Insulin receptor substrate 4	IRS4	biological regulation;cell surface receptor linked signalin	binding;binding, bridging;enzyme binding;insulir	cell part;cytoplasmic part;cytosol;intrace	48	48	48	56.7	56.7	56.7	133.77	0	323.31	7276900000	696	15.5062	0.00333333	O14654;H3BTC1	O14654
Ubiquitin thioesterase ZRANB1	ZRANB1	biological regulation;cell migration;cell motility;cell surfa	binding;catalytic activity;cation binding;cysteine-	cell part;cytoplasm;intracellular membra	38	38	38	57.9	57.9	57.9	80.966	0	323.31	1.4023E+10	953	10.7647	0.0036	Q9UGI0	Q9UGI0
Very-long-chain 3-oxoacyl-CoA reductase	HSD17B12	acyl-CoA biosynthetic process;acyl-CoA metabolic proce	binding;carbohydrate binding;catalytic activity;e	cell part;cytoplasmic part;endoplasmic re	15	15	15	58.3	58.3	58.3	34.324	0	323.31	2622500000	181	10.8233	0.00548387	Q53GQ0;A0A1B0GV93;A0A1B0GVY6;E9PI21;Q53GQ0-2;A0A1B0GW85;A0A1B0GU83	Q53GQ0;A0A1B0GV93;A0A1B0GVY6
Ubiquitin thioesterase OTUB1	OTUB1	biological regulation;cellular macromolecule metabolic p	binding;catalytic activity;cysteine-type peptidase	cell part;cytoplasm;extracellular membra	13	13	13	58.5	58.5	58.5	28.05	0	323.31	1573200000	155	16.959	0.00228571	F5GYN4;J3KR44;Q96FW1;F5H6Q1;F5GYJ8;F5H3F0;Q96FW1-2	F5GYN4;J3KR44;Q96FW1;F5H6Q1;F5GYJ8;F5H3F0;Q96FW1-2
RanBP-type and C3HC4-type zinc finger-containing protein 1	RBCK1	activation of immune response;antigen receptor-mediat	acid-amino acid ligase activity;binding;catalytic a	cell part;intracellular part;LUBAC comple	24	24	12	58.6	58.6	28.8	57.571	0	323.31	8683600000	407	15.6831	0.00133333	Q9BYM8;Q9BYM8-3;G3XAG9;A6PVK0	Q9BYM8;Q9BYM8-3;G3XAG9
Mitochondrial 2-oxoglutarate/malate carrier protein	SLC25A11	alcohol biosynthetic process;alcohol metabolic process;	active transmembrane transporter activity;anior	cell part;cytoplasmic part;integral to men	15	15	15	58.9	58.9	58.9	34.061	0	323.31	1393800000	185	9.35169	0.0176981	Q02978;I3L1P8;Q02978-2	Q02978;I3L1P8;Q02978-2
E3 ubiquitin-protein ligase UBR5	UBR5	biological regulation;catabolic process;cell proliferation;	acid-amino acid ligase activity;binding;catalytic a	cell part;cytoplasm;intracellular membra	136	136	123	59.5	59.5	55.3	309.22	0	323.31	3.6261E+10	1947	18.8659	0.0024	O95071-2;O95071;E7EMW7;E7ET84;H0YAV7;H0YBB4;Q9BWT7-2;Q9BWT7	O95071-2;O95071;E7EMW7
Armadillo repeat-containing X-linked protein 3	ARMCX3	biological regulation;cellular localization;cellular macromolecule localization;cellular process;cellular prote		cell part;cytoplasmic part;integral to men	19	19	19	59.9	59.9	59.9	42.5	0	323.31	1.0466E+10	339	20.2806	0	Q9UH62;H3BV87;H3BM63	Q9UH62
Trifunctional enzyme subunit beta, mitochondrial;3-ketoacyl-CoA thiolase	HADHB	biosynthetic process;carboxylic acid catabolic process;ca	3-hydroxyacyl-CoA dehydrogenase activity;acety	cell part;cytoplasmic part;endoplasmic re	21	21	21	60.4	60.4	60.4	48.879	0	190.09	3110000000	272	14.3935	0.00214286	P55084-2;P55084;F5GZQ3;B5MD38;C9JEY0;C9JE81;C9K0M0	P55084-2;P55084;F5GZQ3;B5MD38
RuvB-like 2	RUVBL2	biological regulation;biosynthetic process;cellular biosy	5'-3' DNA helicase activity;adenyl nucleotide bin	cell part;chromatin;chromatin remodelin	24	24	24	60.5	60.5	60.5	51.156	0	323.31	5305100000	231	12.1255	0.00908571	Q9Y230;Q9Y230-2;M0R0Y3;X6R2L4;M0QXI6;M0R0Z0;A0A1W2PS48	Q9Y230;Q9Y230-2;M0R0Y3
Heme oxygenase 1	HMOX1	anatomical structure formation involved in morphogene	binding;catalytic activity;cation binding;enzyme l	caveola;cell part;cytoplasmic part;cytoso	16	16	16	60.8	60.8	60.8	32.818	0	323.31	8262200000	477	21.7347	0	P09601;B1AHA8	P09601;B1AHA8
Calumenin	CALU	biological regulation;blood coagulation;cell activation;ce	binding;calcium ion binding;cation binding;ion bi	cell part;cytoplasmic membrane-bounde	17	17	3	61.9	61.9	12.4	37.106	0	323.31	1380100000	171	9.77274	0.00984615	O43852;O43852-3;O43852-5;O43852-6;O43852-9;O43852-15;O43852-10;H0Y875;O43852-13;O43852-14;O43852-11;O43852-12	O43852;O43852-3;O43852-5;O43852-6;O43852-9;O43852-15;O43852-10;H0Y875
Reticulocalbin-1	RCN1	anatomical structure development;camera-type eye dev	binding;calcium ion binding;cation binding;ion bi	cell part;cytoplasmic part;endoplasmic re	18	18	18	62.8	62.8	62.8	38.89	0	314.96	1271000000	171	8.04	0.0124681	Q15293;Q15293-2;E9PP27	Q15293;Q15293-2
Stromal cell-derived factor 2-like protein 1	SDF2L1	biological regulation;catabolic process;cellular catabolic process;cellular macromolecule catabolic process		cell part;cytoplasmic part;endoplasmic re	8	8	8	63.3	63.3	63.3	23.598	0	323.31	708540000	82	7.52392	0.0171429	Q9HCN8	Q9HCN8
NADH dehydrogenase [ubiquinone] 1 alpha subcomplex subunit 9, mitochondrial	NDUFA9	cation transport;cellular metabolic process;cellular proc	binding;catalytic activity;coenzyme binding;cofa	cell part;cytoplasmic part;intracellular me	21	21	21	63.4	63.4	63.4	42.509	0	148.47	2021600000	178	15.4744	0.002	Q16795;H3BRM9;F5H0J3;F5GY40	Q16795
Tax1-binding protein 1	TAX1BP1	apoptosis;biological regulation;cell death;cellular proces	binding;cation binding;enzyme binding;ion bindi	cell part;cytoplasmic part;cytosol;extrace	47	47	2	64.7	64.7	2.8	86.256	0	323.31	1.5811E+10	742	13.9115	0.00175	Q86VP1-2;Q86VP1-4;Q86VP1-3;H7C3L5	Q86VP1-2;Q86VP1-4;Q86VP1-3
Dolichol-phosphate mannosyltransferase subunit 1	DPM1	alcohol metabolic process;biosynthetic process;carbohy	alcohol binding;binding;carbohydrate binding;ca	cell part;cytoplasmic part;dolichol-phosp	18	18	18	64.8	64.8	64.8	32.426	0	323.31	4802900000	290	13.4594	0.00331915	Q5QPK2;H0Y368;O60762;Q5QPJ9	Q5QPK2;H0Y368;O60762;Q5QPJ9
E3 ubiquitin-protein ligase RNF31	RNF31	activation of immune response;antigen receptor-mediat	acid-amino acid ligase activity;binding;catalytic a	CD40 receptor complex;cell part;cytoplas	55	55	55	64.9	64.9	64.9	119.65	0	323.31	2.0037E+10	908	11.392	0.00292683	Q96EP0;H0YKX0;Q96EP0-3;A0A0B4J293;Q96EP0-2;H0YM13;H0YMG8;H0YM83;H0YMN0;H0YKI4;H0YNT1;H0YNJ0;H0YMG4;H0YNK5	Q96EP0;H0YKX0;Q96EP0-3;A0A0B4J293
Ubiquitin-associated domain-containing protein 1	UBAC1	cellular macromolecule metabolic process;cellular metabolic process;cellular process;cellular protein meta		cell part;cytoplasm;cytoplasmic part;extr	25	25	25	65.2	65.2	65.2	45.338	0	298.77	2287400000	202	11.1762	0.004	Q9BSL1	Q9BSL1
Mitochondrial import inner membrane translocase subunit TIM14	DNAJC19	anatomical structure development;cellular macromolecule metabolic process;cellular metabolic process;c		cell part;cytoplasmic part;integral to men	9	9	9	65.5	65.5	65.5	12.498	0	95.451	1293200000	51	11.3183	0.00522034	Q96DA6;Q96DA6-2;F2Z3A7;G5E9V2	Q96DA6;Q96DA6-2
Phosphatidylglycerophosphatase and protein-tyrosine phosphatase 1	PTPMT1	biosynthetic process;carbohydrate metabolic process;ca	catalytic activity;hydrolase activity;hydrolase act	cell part;cytoplasmic part;integral to men	12	12	12	68.2	68.2	68.2	22.843	0	223.38	4858700000	144	19.3087	0	Q8WUK0;Q8WUK0-2;E9PQM0;Q8WUK0-3	Q8WUK0;Q8WUK0-2
Ubiquitin carboxyl-terminal hydrolase isozyme L1;Ubiquitin carboxyl-terminal hydrolase	UCHL1	adult behavior;adult locomotory behavior;adult walking	adrenergic receptor binding;alpha-2A adrenergi	axon;cell body;cell part;cell projection;cy	16	16	16	68.6	68.6	68.6	24.824	0	323.31	1.7625E+10	525	7.55064	0.01055	P09936;D6R956;D6RE83;D6R974;D6RF53	P09936;D6R956;D6RE83;D6R974
ATPase WRNIP1	WRNIP1	biological regulation;biosynthetic process;cellular biosy	adenyl nucleotide binding;adenyl ribonucleotide	cell part;cytoplasmic part;intracellular me	40	40	23	69.6	69.6	42	72.132	0	323.31	2.5347E+11	2744	12.2057	0.00224	Q96S55;Q96S55-2;Q96S55-3	Q96S55;Q96S55-2;Q96S55-3
Proliferating cell nuclear antigen	PCNA	anatomical structure development;anatomical structure	binding;catalytic activity;chromatin binding;dam	cell part;centrosome;chromosomal part;	13	13	13	70.1	70.1	70.1	28.768	0	178.91	1370700000	226	9.9007	0.0156538	P12004	P12004
Acylglycerol kinase, mitochondrial	AGK	activation of protein kinase activity;activation of protein	acylglycerol kinase activity;adenyl nucleotide bin	cell part;cytoplasmic part;intracellular me	26	26	26	73	73	73	47.137	0	323.31	1.3462E+10	455	18.049	0.00266667	Q53H12;E9PC15;E9PG39;A0A0G2JLG5;A0A0G2JLD0;Q53H12-2;F8WDD1	Q53H12;E9PC15;E9PG39;A0A0G2JLG5
Ubiquitin carboxyl-terminal hydrolase 5	USP5	biological regulation;catabolic process;cellular catabolic	binding;catalytic activity;cation binding;cysteine-	cell part;cytoplasmic part;intracellular me	58	58	55	73.9	73.9	72.7	93.307	0	323.31	1.3888E+11	2189	12.3136	0.00233333	P45974-2;P45974;F5H571;REV__Q9UPY3-2;REV__Q9UPY3;Q8IWA5-3;Q8IWA5;Q8IWA5-2	P45974-2;P45974
TOM1-like protein 2	TOM1L2	biological regulation;cellular process;cellular response to	binding;clathrin binding;enzyme binding;kinase b	cell part;extracellular membrane-bounde	32	32	32	74	74	74	55.556	0	323.31	1.8002E+10	1094	13.5869	0.00189474	Q6ZVM7;Q6ZVM7-5;Q6ZVM7-2;Q6ZVM7-3;B7Z2U2;F5H3S6;Q6ZVM7-4;K7ENB0;K7EKL6	Q6ZVM7;Q6ZVM7-5;Q6ZVM7-2;Q6ZVM7-3;B7Z2U2;F5H3S6;Q6ZVM7-4
Cytochrome c oxidase subunit 6C	COX6C	biosynthetic process;cellular biosynthetic process;cellul	catalytic activity;cation transmembrane transpo	cell part;cytoplasmic part;integral to men	11	11	11	74.7	74.7	74.7	8.7813	0	56.526	4626100000	112	11.7319	0.00285714	P09669	P09669
Tubulin beta-4A chain	TUBB4A	cell cycle;cell cycle process;cellular component assembly	binding;catalytic activity;GTP binding;GTPase act	axon part;cell body;cell part;cell projectio	26	5	3	75.7	16	10.1	49.585	0	262.94	610440000	105	8.22689	0.0191429	P04350;M0QY85;M0QYM7;M0R278;M0R0X0;M0QY37;M0QX14;M0R042;M0QZL7;M0R1I1	P04350
TIP41-like protein	TIPRL	biological regulation;cell cycle checkpoint;cellular process;cellular response to stimulus;cellular response t		cell part;cytoplasm;intracellular membra	17	17	17	76.8	76.8	76.8	31.444	0	323.31	1.2486E+10	193	7.44222	0.0105854	O75663;O75663-2	O75663;O75663-2
Phospholipase A-2-activating protein	PLAA	biological regulation;cellular lipid metabolic process;cellu	enzyme activator activity;enzyme regulator activ	extracellular membrane-bounded organe	58	58	47	77.1	77.1	64.9	87.156	0	323.31	9.2968E+10	2086	28.9893	0	Q9Y263;E5RIM3;H0YBW4;H0YAU9	Q9Y263;E5RIM3
Ubiquitin carboxyl-terminal hydrolase 13	USP13	autophagy;biological regulation;catabolic process;cell di	binding;catalytic activity;cation binding;cysteine-	cell part;cytoplasmic part;intracellular me	57	54	54	80.1	78.9	78.9	97.326	0	323.31	1.0193E+11	1996	10.7101	0.00252632	Q92995;Q92995-2;H7C5J3;H7C502	Q92995;Q92995-2
Sequestosome-1	SQSTM1	autophagy;biological regulation;catabolic process;cell cc	binding;catalytic activity;cation binding;enzyme l	aggresome;autophagic vacuole;cell part;c	29	29	29	89.3	89.3	89.3	47.687	0	323.31	4.0878E+10	1644	13.692	0.00215385	Q13501;E7EMC7;Q13501-2;E3W990;E9PFW8;D6RBF1;C9J6J8;C9JRJ8	Q13501;E7EMC7;Q13501-2
